# Streptomyces coelicolor Vesicles: Many Molecules To Be Delivered

**DOI:** 10.1128/AEM.01881-21

**Published:** 2022-01-11

**Authors:** Teresa Faddetta, Giovanni Renzone, Alberto Vassallo, Emilio Rimini, Giorgio Nasillo, Gianpiero Buscarino, Simonpietro Agnello, Mariano Licciardi, Luigi Botta, Andrea Scaloni, Antonio Palumbo Piccionello, Anna Maria Puglia, Giuseppe Gallo

**Affiliations:** a Department of Biological, Chemical and Pharmaceutical Sciences and Technology, University of Palermo, Palermo, Italy; b Proteomic and Mass Spectrometry Laboratory, ISPAAM, Consiglio Nazionale delle Ricerche Napoli, Italy; c Department of Biology, University of Florence, Sesto Fiorentino, Italy; d Advanced Technologies Network Center, University of Palermo, Palermo, Italy; e Department of Physics and Chemistry, University of Palermo, Palermo, Italy; f Department of Engineering, University of Palermo, Palermo, Italy; University of Tennessee at Knoxville

**Keywords:** *Streptomyces*, actinomycetes, antibiotics, electron microscopy, membrane vesicles, metabolomics, proteomics

## Abstract

Streptomyces coelicolor is a model organism for the study of *Streptomyces*, a genus of Gram-positive bacteria that undergoes a complex life cycle and produces a broad repertoire of bioactive metabolites and extracellular enzymes. This study investigated the production and characterization of membrane vesicles (MVs) in liquid cultures of S. coelicolor M145 from a structural and biochemical point of view; this was achieved by combining microscopic, physical and -omics analyses. Two main populations of MVs, with different sizes and cargos, were isolated and purified. S. coelicolor MV cargo was determined to be complex, containing different kinds of proteins and metabolites. In particular, a total of 166 proteins involved in cell metabolism/differentiation, molecular processing/transport, and stress response were identified in MVs, the latter functional class also being important for bacterial morpho-physiological differentiation. A subset of these proteins was protected from degradation following treatment of MVs with proteinase K, indicating their localization inside the vesicles. Moreover, S. coelicolor MVs contained an array of metabolites, such as antibiotics, vitamins, amino acids, and components of carbon metabolism. In conclusion, this analysis provides detailed information on S. coelicolor MVs under basal conditions and on their corresponding content, which may be useful in the near future to elucidate vesicle biogenesis and functions.

**IMPORTANCE** Streptomycetes are widely distributed in nature and characterized by a complex life cycle that involves morphological differentiation. They are very relevant in industry because they produce about half of all clinically used antibiotics, as well as other important pharmaceutical products of natural origin. Streptomyces coelicolor is a model organism for the study of bacterial differentiation and bioactive molecule production. S. coelicolor produces extracellular vesicles that carry many molecules, such as proteins and metabolites, including antibiotics. The elucidation of S. coelicolor extracellular vesicle cargo will help us to understand different aspects of streptomycete physiology, such as cell communication during differentiation and response to environmental stimuli. Moreover, the capability of these vesicles for carrying different kinds of biomolecules opens up new biotechnological possibilities related to drug delivery. Indeed, decoding the molecular mechanisms involved in cargo selection may lead to the customization of extracellular vesicle content.

## INTRODUCTION

Bacteria communicate and coordinate their activities through chemical signals that are often secreted into the extracellular environment (1–3). In this context, the release of extracellular vesicles (EVs) might play a fundamental role in microbial life as a universally conserved mechanism of intercellular communication. EVs are non-replicative, nano-sized, spherical particles delimitated by a lipid bilayer. They contain various macromolecules, such as nucleic acids, lipids, and proteins (i.e., enzymes and toxins), and small molecules, such as cellular metabolites and bioactive molecules (i.e., antibiotics) (4–6). EV-mediated secretion and delivery presents several advantages: (i) the lipid bilayer encasing the vesicular cargo confers protection from environmental degradation; (ii) embedded biomolecules are maintained at high concentration; (iii) it facilitates the delivery of molecules that would otherwise be excluded from entering target cells because of their size, charge, or hydrophobicity (7–9). Accordingly, it has been proposed that vesicular secretion represents a new secretion system, also called secretion system type zero (7).

Most of the studies performed on bacterial vesicles have been carried out on Gram-negative bacteria, where EVs originate from the outer membrane; therefore, they are often referred to as outer membrane vesicles (OMVs) (10). The population of vesicles released from bacteria is heterogeneous in size, and limited knowledge is available regarding the possible correlation between size and cargo (11). Interestingly, a heterogeneous population of OMVs was identified in Helicobacter pylori, with two principal subpopulations being characterized by different content and differing ability to enter host cells (11). Gram-positive extracellular membrane vesicles (MVs) have only attracted attention in recent years: for a long time, Gram-positive bacteria were considered unable to shed EVs due to their lack of an outer membrane layer and the presence of a thick cell wall (4, 6). Nevertheless, the release of MVs into the surrounding environment has been observed in Gram-positive bacterial species belonging to the phyla Firmicutes and Actinobacteria. For example, MVs isolated from *Streptomyces* Mg1 culture supernatants were shown to carry the linear polyketide linearmycins. Interestingly, a linearmycin-deficient strain of *Streptomyces* Mg1 was demonstrated to be defective for the production of MVs, suggesting a deep relationship between antibiotic biosynthesis and the physiology of cellular membranes (5). Moreover, Fröjd et al. (2019) observed the release of MVs from the tips of vegetative hyphae of Streptomyces venezuelae, for which treatment with vancomycin-blocking peptidoglycan synthesis was shown to lead to a high frequency of vesicle extrusion (12). All these findings suggest that MV biogenesis and functions are related to the streptomycete life cycle, particularly with regard to physiological differentiation and growth, as related to antibiotic production and cell-wall biosynthesis, respectively. In any case, the exact biological role of streptomycete MVs is still elusive and a complete repertoire of their cargo has not yet been elucidated.

Streptomyces coelicolor is a model of study widely used to investigate the regulation of microbial morphological differentiation and the production of bioactive molecules (13). S. coelicolor is characterized by a complex life cycle, encompassing its transition from a vegetative mycelium to reproductive aerial hyphae that eventually differentiate in chains of spores. At the level of regulatory cascades of gene expression, this morphological differentiation is related to the production of different antibiotics, including actinorhodin (ACT), undecylprodigiosin, and calcium-dependent antibiotics (14). Intercellular communication is an important aspect of *Streptomyces* morphological differentiation, which is affected by a number of external factors (15, 16). When grown on complete solid medium, S. coelicolor produces evident, blue-pigmented exudates (also known as blue droplets, for the presence of blue-pigmented actinorhodin) that also contain MVs with high concentrations of various proteins. Based on scientific literature and/or sequence homology, these proteins were associated with different processes, such as the uptake of inorganic/organic phosphate, iron ions, and certain carbon sources, as well as energy metabolism, redox balance, and defense against oxidants (17).

EV isolation and purification constitute a crucial step for their subsequent characterization, which requires a proper quantity of vesicles. Usually, EVs are prepared from liquid cultures after suitably long cultivation times (18). Since the industrial-scale production of bioactive molecules in *Streptomyces* is mostly performed in liquid medium, it would be interesting to study the production of MVs in liquid cultures. In this study, we report the production and the physicochemical and biochemical characterization of MVs derived from S. coelicolor grown in liquid cultures, in a strain which is known to produce molecules with various biological functions.

## RESULTS

### S. coelicolor produces MVs in liquid culture.

**FIG 1:**
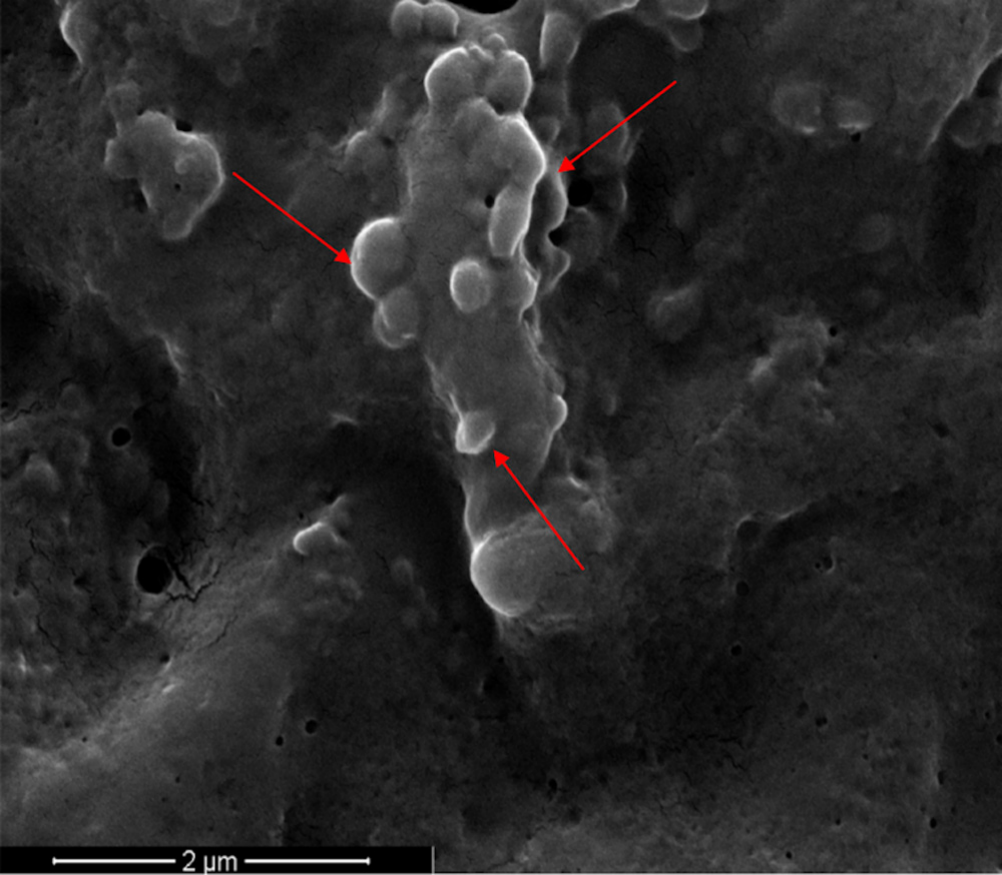
Scanning electron microscopy (SEM) image of S. coelicolor hyphae. Image was acquired after 6 days of bacterial growth in a liquid medium. Red arrows indicate some of the emerging MVs on the surface of bacterial hyphae.

**FIG 2:**
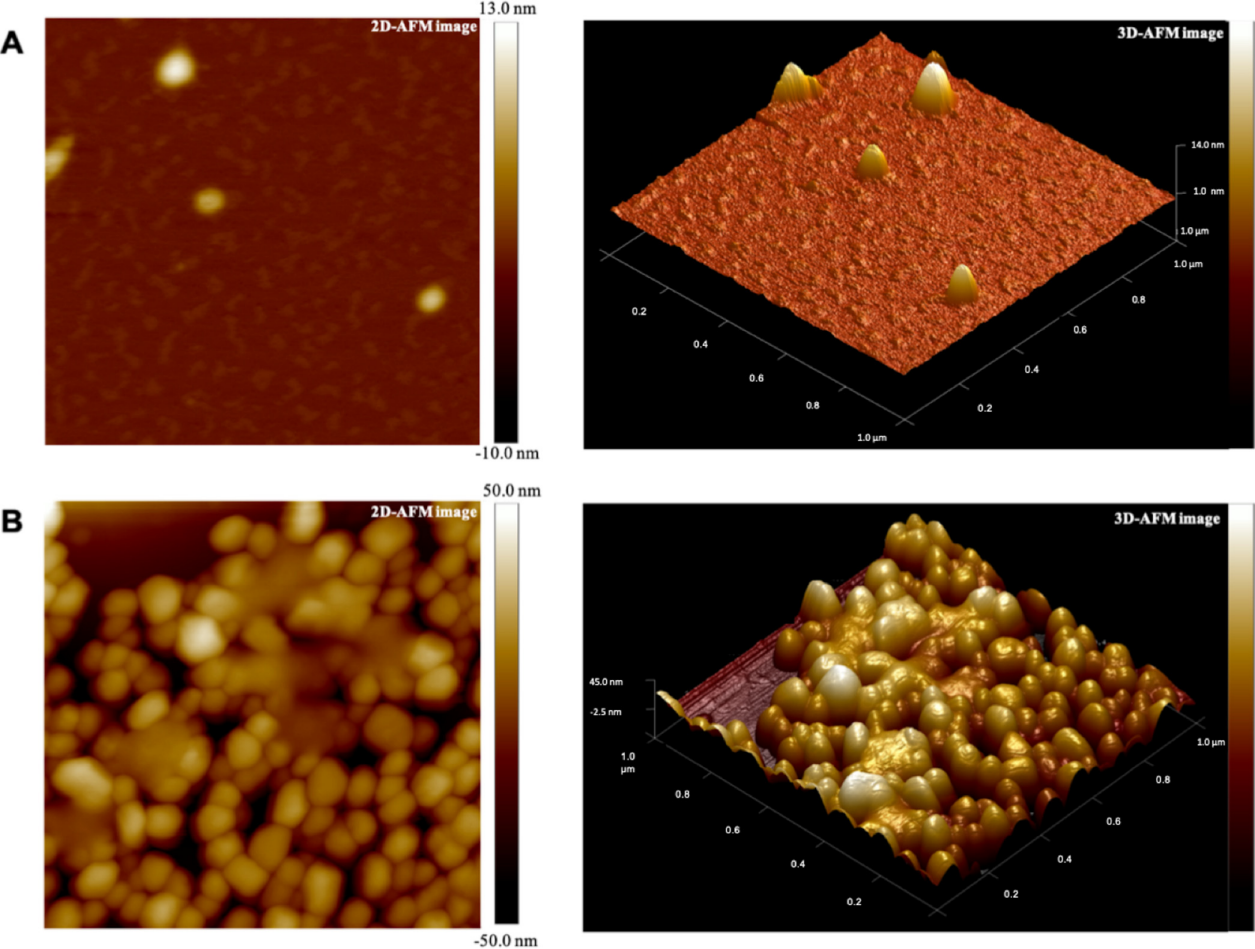
2D (left) and 3D (right) representations of the atomic force microscopy (AFM) images of S. coelicolor MVs from fractions F3 (A) and F4 (B).

**FIG 3:**
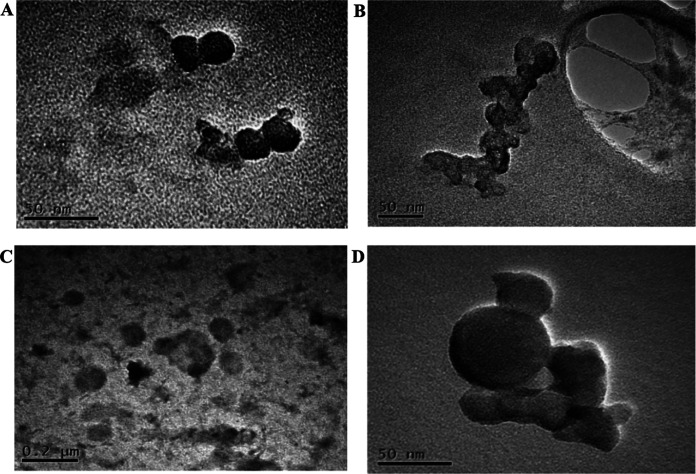
Transmission electron microscopy (TEM) micrographs of S. coelicolor MVs from fractions F3 (A and B) and F4 (C and D).

**FIG 4:**
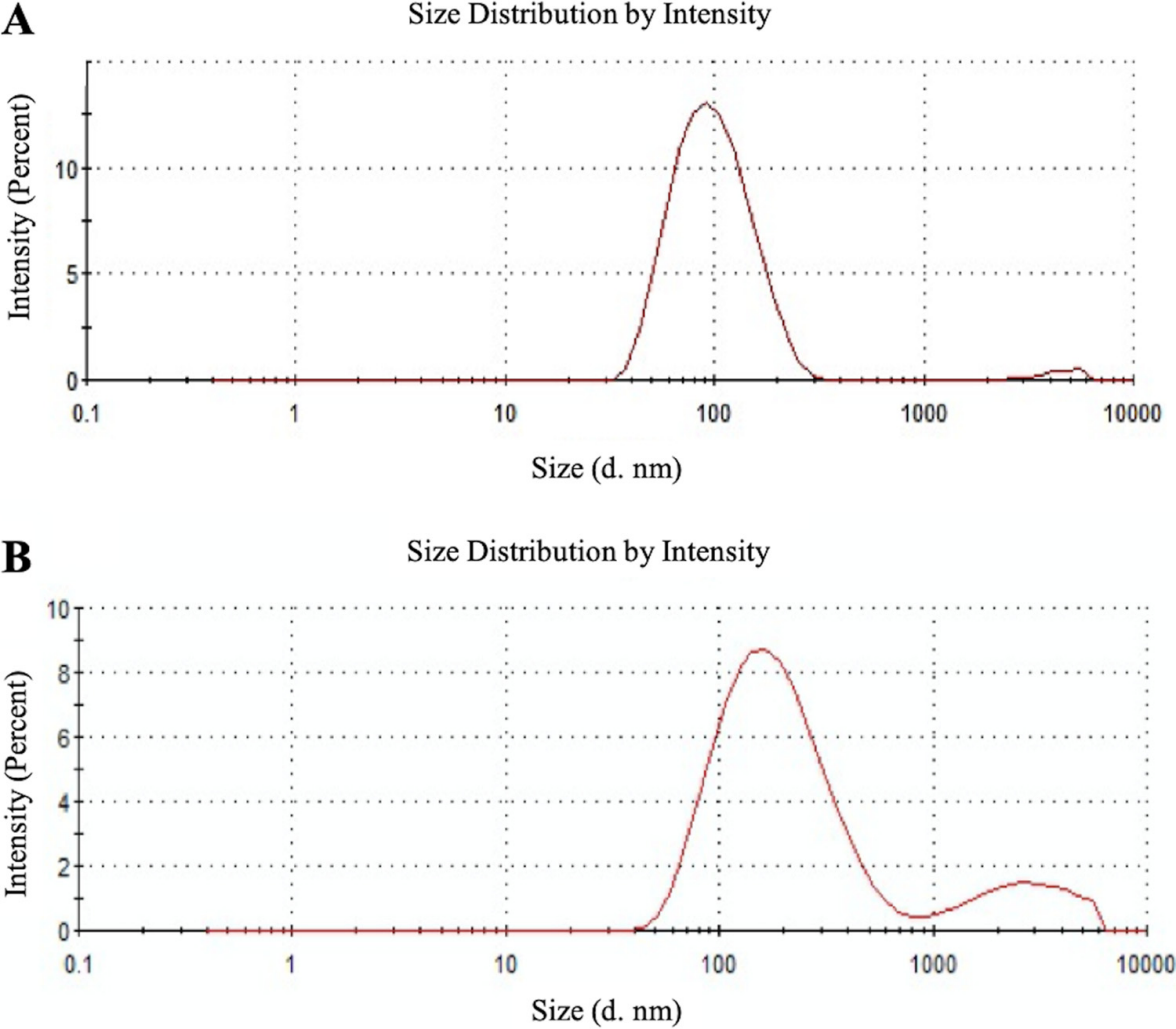
Dynamic light scattering (DLS) analysis of S. coelicolor MVs. MVs from fractions F3 (A) and F4 (B) showed average sizes of 100 and 200 nm, respectively.

Previous observations of other Gram-positive bacteria (19, 20) suggest significant production of MVs during the late exponential and/or stationary phases of microorganism growth, and reduced or negligible MV generation at the corresponding early stages. Accordingly, the release of MVs by S. coelicolor was investigated after 6 days of growth in a liquid minimal medium, corresponding to late stationary growth stages; this time point is characterized by the presence in cultures of the blue actinorhodin antibiotic biosynthesized by the bacterial strain. In this experimental condition, putative MVs were detected on the surface of hyphae, thus demonstrating that vesicles bud off from cells during late growth stages (Fig. 1). Therefore, at this growth time, MVs were isolated from S. coelicolor cultivations and further purified from the culture supernatant through sequential filtration, centrifugation, and ultracentrifugation. To this purpose, we set up a dedicated protocol for Gram-positive bacteria that resolves MVs in six ultracentrifugation fractions, designated F1 through F6 (21). This protocol showed that only specific fractions, namely F3 and F4, contain MVs, which were also determined as pure. Accordingly, this observation was also confirmed in the case of S. coelicolor MVs. Indeed, the F1 through F6 fractions were analyzed by 12% T SDS-PAGE, with the MVs present in the F3 and F4 fractions showing the most abundant and complex protein representation (Fig. S1). On the other hand, 12% T SDS-PAGE analysis of F1 through F6 fractions, obtained from cultivations at the early stages (24 and 48 h), did not reveal any protein patterns. Therefore, MVs of the F3 and F4 fractions, collected from 6-day-old cultivations, were then analyzed by atomic force microscopy (AFM), revealing sphere-shaped vesicles (Fig. 2). The spherical shape of MVs in F3 and F4 fractions was also confirmed by transmission electron microscopy (TEM), which also showed an electron-dense luminal content (Fig. 3). The aforementioned content was consistent with the notion that vesicles contained proteins and nucleic acids (20). Interestingly, dynamic light scattering (DLS) analysis revealed that these two fractions contained vesicles of different sizes. In particular, the MVs in fraction F3 had an average size of 100 nm (polydispersity index [PDI] value, 0.182), while their counterparts in fraction F4 were larger, with an average size of 200 nm (PDI value, 0.370) (Fig. 4). The average size of the MVs agreed with TEM analysis, considering the volumetric shrinkage due to evaporation of the medium/solvent. Diameter variations of isolated MVs might indicate different biological roles and different biogenesis (22).

### Proteomics of S. coelicolor MVs.

**FIG 5:**
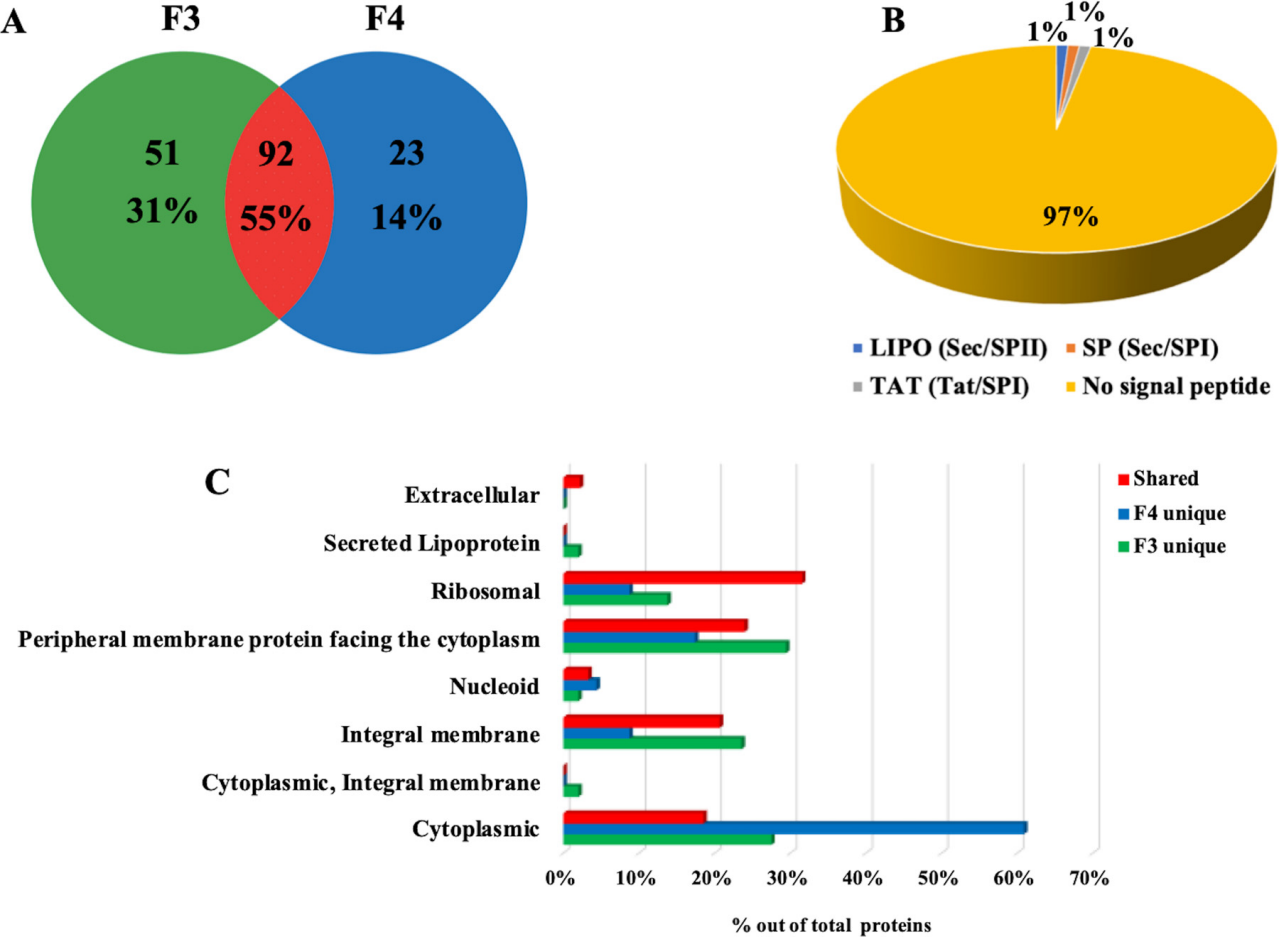
S. coelicolor MVs are enriched in proteins. (A) Venn diagram showing the number of proteins present in MVs from fractions F3 and F4, as identified by SDS-PAGE, proteomics, and bioinformatics. Percentage values refer to the total number of all proteins identified. (B) Prediction of the presence of signal peptide cleavage sites in proteins identified in bacterial MVs. Sec signal peptide (Sec/SPI; gray); lipoprotein signal peptide (Sec/SPII; blue); Tat signal peptide (Tat/SPI; orange); no signal peptide (yellow). (C) Predicted subcellular localization of proteins exclusively present in either F3 or F4 MVs (“F3 unique” and “F4 unique”) or shared between the F3 and F4 MVs (“shared”). Percentage values refer to the total number of proteins identified in each fraction or shared.

MVs in F3 and F4 fractions were analyzed using SDS-PAGE coupled with nanoLC-Q-Orbitrap-MS/MS analysis and bioinformatics. A total of 166 protein species were identified (Table S1), with 92 proteins (55%) being shared among MVs present in both F3 and F4 fractions, 51 (31%) exclusively present in F3 MVs, and 23 (14%) only observed in F4 MVs (Fig. 5A). Most secreted proteins and surface proteins are known to have a signal peptide sequence in their N-terminus, marking them for correct sorting to specific cellular domains. Thus, the signal peptide predictor SignalP 5.0 was used to categorize proteins identified in S. coelicolor MVs according to the presence of putative signal peptide sequences; this analysis revealed that only 3% of vesicle proteins contained a signal peptide (Fig. 5B). For a more detailed characterization, the identified proteins were then annotated for their subcellular localization using the Subcellular Topology of Polypeptides in *Streptomyces* (SToPSdb) database (23). This analysis demonstrated that proteins annotated as extracellular were poorly represented, while the most-represented proteins belonged to the following categories: (i) “peripheral membrane proteins facing the cytoplasm” (29%), for proteins present exclusively in F3 MVs; (ii) “cytoplasmic” (61%), for proteins present exclusively in F4 MVs; or “ribosomal” (32%), for proteins shared between the MVs of both F3 and F4 fractions (Fig. 5C). Several identified proteins were already classified as moonlighting proteins (i.e., proteins having multiple diverse and unrelated functionalities besides their principal biological role), such as chaperone protein DnaK, enolase 1 (Eno1), 60 kDa chaperonin 1 (GroEL1), 60 kDa chaperonin 2 (GroEL2) and superoxide dismutase (SodF1) (Table S1) (24, 25). The functions of identified proteins were categorized according to the KEGG metabolic database; most of them were involved in different functions, as expected in the case of moonlighting proteins. The KEGG Mapper tool was then used to visualize the metabolic/functional pathways of vesicular proteins. This analysis revealed that proteins involved in central carbohydrate metabolism, energy metabolism, and oxidative phosphorylation were common to both vesicle fractions. Conversely, proteins involved in peptidoglycan biosynthesis, fructose/mannose metabolism, and biosynthesis of type II polyketide backbone were specific to vesicles present in fraction F3, while proteins involved in amino acid metabolism and purine/pyrimidine metabolism were exclusive to vesicles present in F4 (Fig. S2).

The S. coelicolor MV proteome also included a set of stress response proteins, with some being shared between the two MV fractions; e.g., DNA integrity scanning protein (DisA), catalase-peroxidase (CpeB), bacterioferritin (Bfr), SodF1, DnaK, GroEL2, GroEL1, Pup-protein ligase (PafA), and polyribonucleotide nucleotidyltransferase (Pnp). Other stress response proteins were fraction-specific, such as ATP-dependent Clp protease proteolytic subunit 2 (ClpP2), cold shock protein ScoF, mycothiol acetyltransferase, Fe^3+^ ion import ATP-binding protein FbpC (present in F3 MVs), and ATP-dependent Clp protease proteolytic subunit 1 (ClpP1; present in MVs from fraction F4) (26–36). Many of them, such as ClpP1, ClpP2, DnaK, GroEL1, GroEL2, Pnp, S-adenosylmethionine synthase (MetK), glutamine synthetase (GlnA), aminopeptidase probable cytosol aminopeptidase (PepA), and anti-sigma-B factor antagonist (BldG), have already been reported to play a role in bacterial morpho-physiological differentiation (Table S1) (35, 37–40).

### The luminal proteome of S. coelicolor MVs.

**FIG 6:**
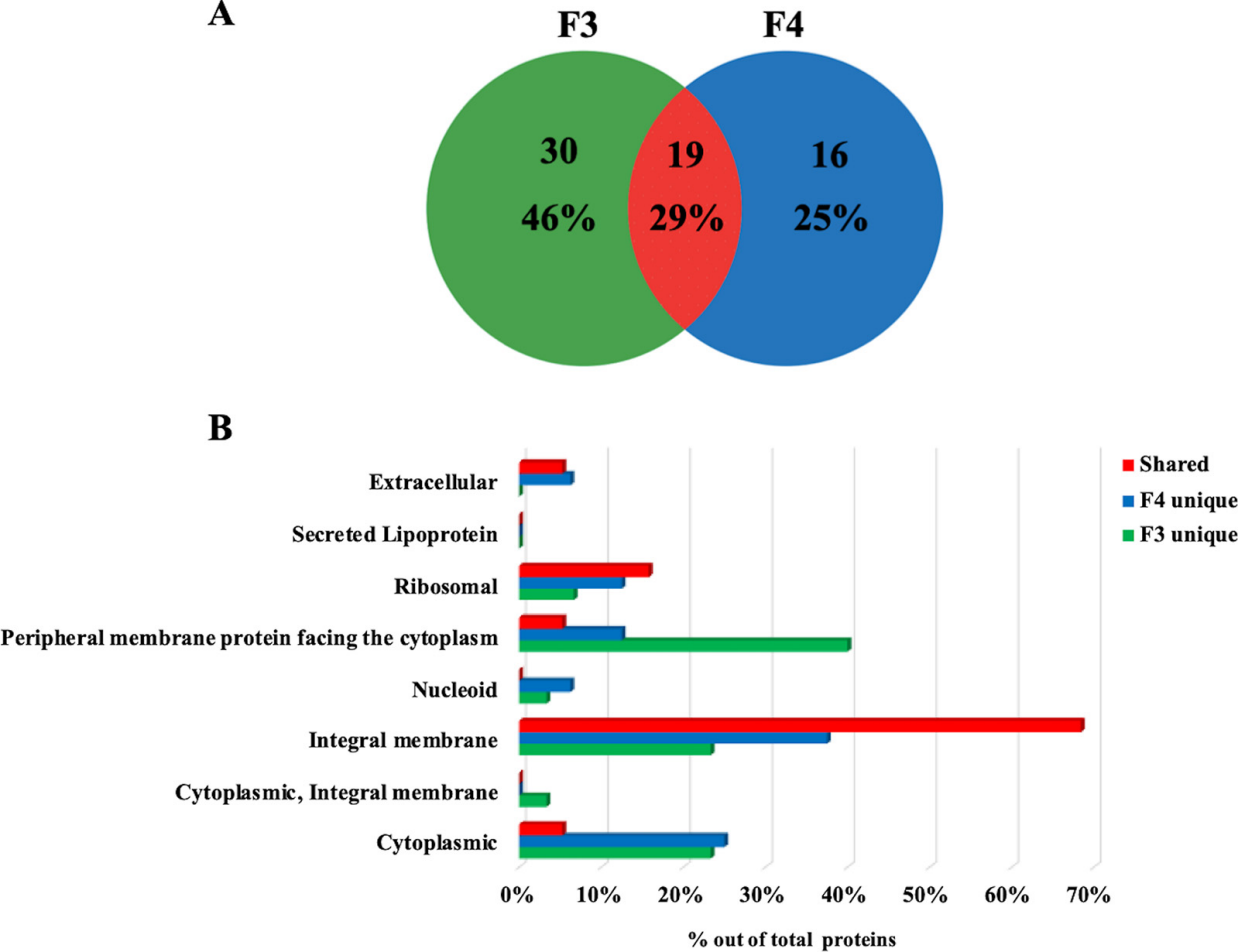
Luminal proteins of S. coelicolor MVs. (A) Venn diagram showing the number of luminal proteins present in F3 and F4 MVs and the number that coincided. Percentage values refer to the total number of all identified proteins. (B) Predicted subcellular localization of proteins exclusively present in F3 or F4 MVs (“F3 unique” and “F4 unique”) or shared between the F3 and F4 MVs (“shared”). Percentage values refer to the total number of proteins identified in each fraction or shared.

In order to define those proteins present in vesicle lumen as well as the components possibly associated with the surface of MVs, MVs were treated with proteinase K (PK) to remove surface-accessible species (“shaving”). Since PK cannot penetrate the membrane of MVs, and DLS analysis revealed that PK treatment did not change MV integrity during the whole experimental period, it is most likely that the proteins susceptible to PK action were localized on the surface of vesicles (41). PK-treated and untreated MVs from fractions F3 and F4 were analyzed in parallel by 12% T SDS-PAGE coupled with nanoLC-Q-Orbitrap-MS/MS and bioinformatics (Fig. S3). In both cases, proteins were identified based on the assignment of multiple (at least two) tryptic peptides deriving from components with a mass of >6 kDa. A comparison between the protein catalogues of PK-treated and untreated MVs using the Normalized Spectral Abundance Factor (NSAF) ratio (42) was carried out. This experiment allowed a tentative identification of internal vesicle proteins (present both in shaved and unshaved MVs) and surface-localized vesicle proteins (present in unshaved MVs and absent in shaved MVs). Sixty-five proteins were classified as internal proteins (Table S1); among them, 19 proteins were common to MVs from both the F3 and F4 fractions, while the other ones were fraction-specific (Fig. 6A). In particular, the most represented proteins belonged to the following categories: (i) “peripheral membrane proteins facing the cytoplasm” (40%) for proteins present exclusively in F3 MVs, and (ii) “integral membrane” for proteins present exclusively in F4 MVs (38%) and for proteins shared between the MVs of both F3 and F4 fractions (68%) (Fig. 6B). Shared proteins included Bfr, spore associated protein A (SapA), SodF1, cytochromes (QcrB, QcrA, CtaD1, CtaC), and secretion system proteins (SecF, SecY).

### Metabolomics of S. coelicolor MVs.

**FIG 7:**
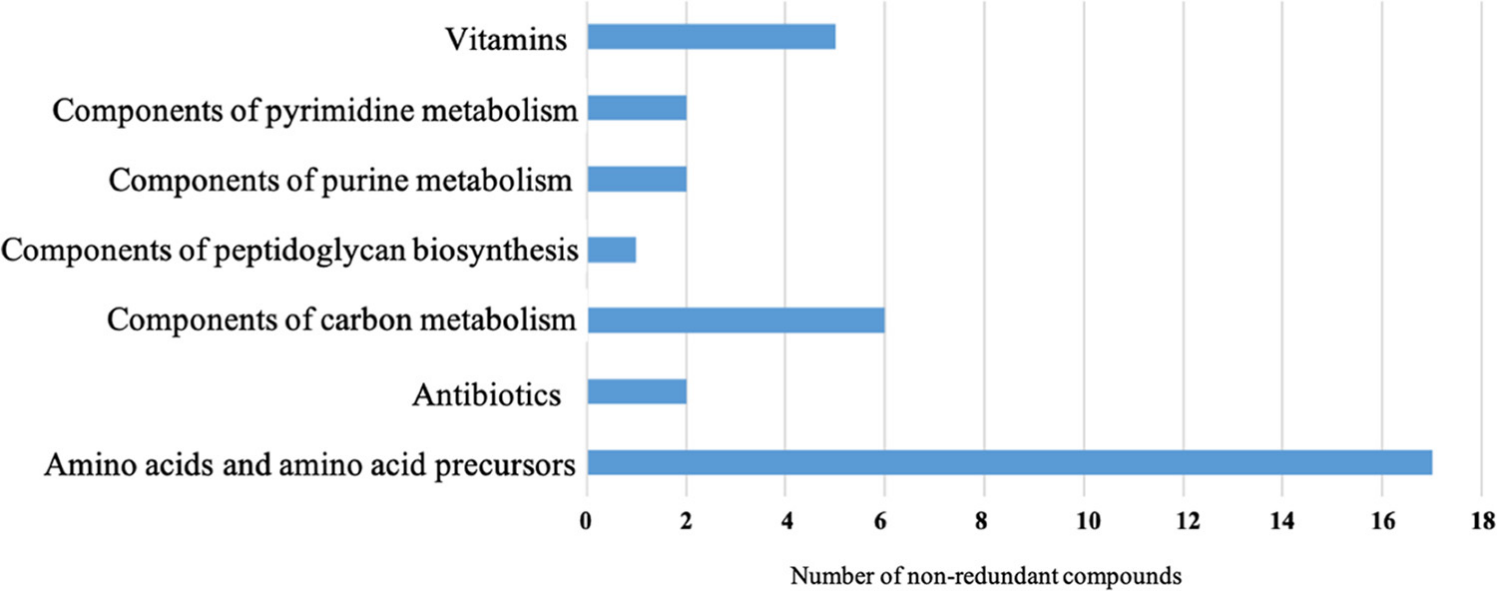
Classes of metabolites identified in S. coelicolor MVs from fractions F3 and F4. The *x* axis represents the number of non-redundant metabolites for each molecular category.

**FIG 8:**
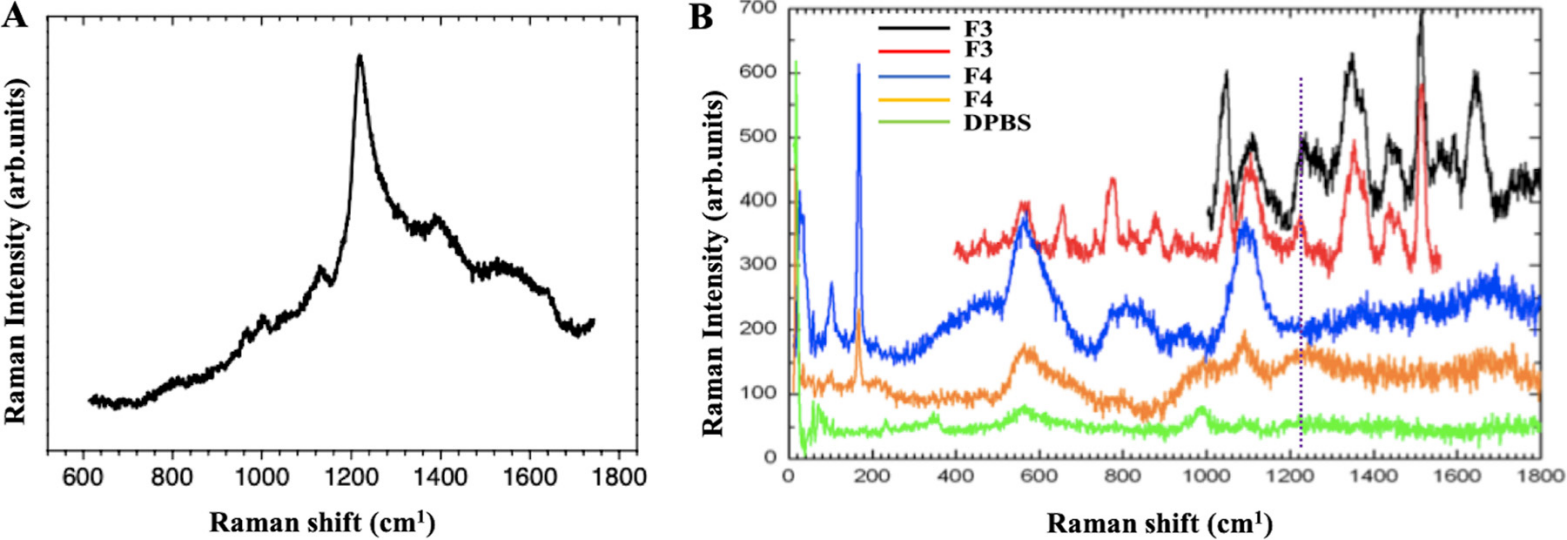
Raman spectra of actinorhodin (A) and MVs from fractions F3 and F4 (B). F3 and F4 MVs were reported as duplicates and DPBS as the negative control.

A targeted metabolomic analysis was performed to identify the metabolic profile of S. coelicolor MVs. Ninety-nine metabolites were detected in both MV fractions (Table S3), and, among them, 35 were suitably abundant (i.e., >0.0005 ppm) to perform an analysis for differential representation. Metabolites were categorized, according to the KEGG compound database, into the following categories: (i) amino acids and amino acid precursors, (ii) vitamins, (iii) components of carbon metabolism, components of (iv) pyrimidine and (v) purine metabolism, (vi) antibiotics, and (vii) components of peptidoglycan biosynthesis (Fig. 7). Comparative quantitative metabolomic analysis showed that 10 metabolites (Table 1) were more abundant (relative abundance of ≥1.3-fold) in MVs from fraction F3 than in those from F4. This is also the case for the antibiotic actinorhodin, which showed amounts 2.1-fold higher in F3 MVs than in F4 MVs, as confirmed by Raman spectroscopy analysis. In fact, spectroscopic measurements detected the specific peak at 1,200 cm^−1^ in MVs from fraction F3, which was also detected in an actinorhodin extract used as reference (Fig. 8). By contrast, this specific signal was not detected in MVs from fraction F4, probably because it occurred below the detection limit of Raman spectroscopy. Additionally, untargeted metabolomic analysis performed on MVs from fraction F3 revealed the presence of other bacterial metabolites, such as streptorubin B, medelamine C, and furaquinocin D (Table S4).

**TABLE 1:** Differentially abundant metabolites in S. coelicolor MVs as defined by targeted metabolomics^*a*^

Metabolite	KEGG ID	Functional classes	Concentration (ppm)		F3 MVs vs. F4 MVs fold change^*b*^
			F3 MVs	F4 MVs	
Phenylalanine	C00079	Amino acids, amino acid precursors	0.049	0.0138	3.6
Isoleucine	C00407	Amino acids, amino acid precursors	0.0245	0.016	1.5
Leucine	C00123	Amino acids, amino acid precursors	0.0183	0.01025	1.8
Serine	C00065	Amino acids, amino acid precursors	0.0044	0.00285	1.5
Tryptophan	C00078	Amino acids, amino acid precursors	0.01525	0.01085	1.4
Actinorhodin	C06691	Antibiotics	0.1599	0.0772	2.1
Undecylprodigiosin	C12023	Antibiotics	0.00705	0.00515	1.4
Glucose-6-phosphate	C00092	Carbon metabolism components	0.0645	0.05115	1.3
Folate	C00504	Vitamins	0.0095	0.00405	2.3
Vitamin B_12_	C05776	Vitamins	0.00085	0.00045	1.9

a Compound name, KEGG ID, functional classification, and abundance profiles of identified metabolites are reported.

b Fold change for each metabolite was calculated by normalizing the amount of F3 fraction to that of its F4 counterpart.

Finally, proteomic and metabolomic data were used to build up a comprehensive metabolic network for S. coelicolor MVs. Interestingly, many metabolites belonged to metabolic pathways associated with the vesicular proteins identified in this study, confirming the consistency of the obtained results (Fig. S2).

## DISCUSSION

This study originally demonstrates the release of protein-carrying MVs by S. coelicolor cultivated in a liquid minimal medium. In particular, MVs were observed during late bacterial growth stages, in perfect agreement with other studies reporting that MVs from Gram-positive bacteria are generally available for significant detection/purification only during the late exponential or stationary growth phases (19, 20). In particular, scanning electron microscopy (SEM) images showed hyphae of 6-day-old cultures with multiple protrusions, which were similar to those already reported for other Gram-positive bacteria such as Bacillus subtilis and Lactobacillus reueteri (43, 44). S. coelicolor MVs were further characterized from a structural and biochemical point of view, taking advantage of combined microscopical, physical, proteomic, and metabolomic procedures.

Two main populations of MVs, namely F3 MVs and F4 MVs, were isolated and purified using a dedicated ultracentrifugation-based protocol. The size of MVs was determined by DLS analysis, with F3 and F4 MVs having average sizes of 100 and 200 nm, respectively. Diameter heterogeneity of isolated MVs might indicate different biological roles and/or different biogenesis.

The morphology of MVs was further investigated by microscopy analyses. AFM images showed the spherical shape of both above-mentioned vesicle populations, and TEM analysis confirmed these characteristics. Moreover, the latter analysis revealed an electron-dense luminal content for both F3 and F4 vesicles, suggesting they behave as bioactive carriers.

The size distribution among MVs and OMVs is similar; however, it is increasingly evident that bacteria produce heterogeneous populations of vesicles with various sizes, densities, and cargos. For example, B. subtilis releases a heterogeneous population of MVs both in term of size and electron density, suggesting a potential quantitative and qualitative correlation with cargo. Moreover, other members of the Firmicutes phylum, such as Staphylococcus spp., Streptococcus pneumoniae, and Listeria monocytogenes, release MVs with a size range of 20 to 150 nm, while other Gram-positive bacteria, such as *Bacillus* spp. and Clostridium perfringens, produce MVs ranging from 20 to 400 nm (4, 11, 43, 45).

Proteomic analysis of F3 and F4 MVs disclosed a complex protein composition with a total of 166 identified protein species. About half (55%) of the identified proteins were common to both F3 and F4 MVs, whereas other components were specific for each type of vesicles. Interestingly, bioinformatic analysis revealed that 97% of the identified proteins do not contain the signal peptide for subcellular localization in their polypeptide precursor sequence. Dedicated bioinformatic analysis also demonstrated that S. coelicolor MVs comprise an array of proteins mostly assigned to the membrane and cytoplasm localizations, with a relevant abundance of ribosomal proteins as well; conversely, proteins annotated as extracellular components were poorly represented. In the light of recent findings suggesting more than one possible function for extensively studied proteins (moonlighting feature), one possible explanation for the presence of cytoplasmic proteins (e.g., DnaK, GroEL1, GroEL2, Eno1, and SodF1) in MVs is that some of them are moonlighting proteins, which have been widely described as having multiple simultaneous locations and performing multiple biological functions (23, 46). In opportunistic and pathogenic bacteria, many of these moonlighting cytosolic proteins act as adhesins or invasins, promoting attachment to and/or entry into host cells (47). For example, the cytoplasmic chaperon proteins GroEL and DnaK, which were primarily known to play a role in protein folding, are relocated to the cell surface or are secreted during invasion (48). Another very intriguing prototypical moonlighting protein is enolase, which is often found in vesicles produced by both Gram-positive and Gram-negative bacteria; indeed, it can act as bacterial adhesin binding host proteins, and plays a role in infections (25, 49–51). Hence, the presence of Eno1 on the surface of S. coelicolor MVs, as demonstrated by PK treatment, might suggest a possible role of this protein in interacting with target cells. In any case, the biological role of S. coelicolor MVs is still elusive and functional study should be performed to elucidate the roles of protein and metabolite cargos in these vesicles. The dedicated construction of knock-in and/or knockout S. coelicolor strains for genes encoding moonlighting proteins may unveil the functional role of these components in *Streptomyces* cell communication.

Identified proteins were categorized by function according to the KEGG metabolic database, and bioinformatic analyses were performed to construct the metabolic pathways of proteins contained in S. coelicolor MVs. Interestingly, some metabolic pathways were common to both MV populations (i.e., central carbohydrate metabolism, energy metabolism and oxidative phosphorylation), while some pathways were specific for MVs from fraction F3 (i.e., peptidoglycan biosynthesis, fructose and mannose metabolism, and biosynthesis of type II polyketide backbone) or MVs from fraction F4 (i.e., amino acid, purine, and pyrimidine metabolism). These data suggested that the MV populations present in fractions F3 and F4 might exert different functional roles and/or be produced in different developmental stages of the S. coelicolor life cycle. This hypothesis is supported by studies on both Gram-negative and Gram-positive bacteria. In fact, a correlation between growth stages, vesicle release, and content has been demonstrated for Pseudomonas aeruginosa, H. pylori, and B. subtilis (19, 52, 53). For example, B. subtilis releases MVs during both the sporulation phase and the vegetative phase, with corresponding vesicular proteomes that are qualitatively and quantitatively different (19).

The proteome of S. coelicolor MVs also included many stress response proteins, namely GroEL1, GroEL2, DnaK, ClpP1, and ClpP2, which are also known to play important roles in bacterial morpho-physiological differentiation. Many authors suggested that the production of vesicles can be considered as a bacterial response to stress conditions (54–56); in S. coelicolor, experimental evidence has been reported for a strong correlation between stress signals and morphological differentiation (31, 35).

The treatment of MVs with PK allowed the identification of 65 proteins—most of them classified as membrane proteins by bioinformatics—that are localized in the luminal portion of S. coelicolor vesicles. Among the most interesting findings are Bfr, SapA, SodF1, cytochromes, and some Sec system proteins. Other studies have demonstrated that many of these proteins constitute the protein cargo of MVs in other organisms (19, 57–59), although they have not detailed their sublocalization in vesicles, whether in the luminal or external region. Bfr and SodF1 are cytosolic proteins involved in the oxidative stress response (60, 61). Thus, their association with vesicles could serve as a defense against stressful environmental conditions. SapA is secreted in liquid cultures of S. coelicolor but its function is unknown under this growth condition (62), so it would be interesting to study its role in vesicles. Specific cytochromes are integral membrane proteins, strengthening the hypothesis that they may have a role in extracellular electron transport in vesicles (10, 58).

Metabolic analyses underlined a complex cargo in S. coelicolor MVs, which includes amino acids and their precursors, vitamins, components of purine and pyrimidine metabolism, components of carbon metabolism, and antibiotics. In particular, the presence of the antibiotics actinorhodin and undecylprodigiosin in S. coelicolor MVs suggest that the corresponding cargos reflect one of the most peculiar features (i.e., bioactive molecule production) of the strain that produces these vesicles. For example, MV cargos of some pathogenic bacteria have already been reported to include virulence factors (4, 63, 64). Thus, it is tempting to speculate that MV function relies on intra- and inter-species communications, which could be inferred by cargo components that, depending on their nature, may deliver cell-specific signal(s) in the environment. In any case, this interesting aspect deserves further studies to shed light on the possible mechanisms that could play key roles in the life cycle of this complex prokaryote.

Little is known about the biogenesis of MVs, and the release of vesicles through the thick cell wall is still disputed. Although it has been demonstrated, in some Gram-positive bacteria, that MVs can arise from dying cells, it is likely that cell death-independent mechanisms may also exist (65). This hypothesis is supported by experimental evidence on MVs from Bacillus anthracis proving the differential enrichment of diverse engulfed molecules under different conditions (66), which suggests the existence of dedicated mechanisms for regulating and controlling vesicle cargo sorting. In *Streptomyces* strains, an intriguing hypothesis may be consistent with the possible role of MVs in exporting bioactive molecules and proteins involved in cell communication/interactions; this could be inferred from the presence of metabolites such as actinorhodin and proteins such as DnaK, Eno1, AfsQ2, Bfr, and Sec system proteins.

Previously reported findings also suggest the possibility of exploiting MV production to selectively deliver metabolites in culture medium without risking putative degradation events, thanks to the protection, provided by the corresponding membranes, against eventual external degrading enzymes. In this perspective, the mechanistic elucidation of MV production by microbial strains cultivated in liquid media could be biotechnologically exploited. Indeed, it is reasonable to imagine that future metabolic engineering strategies might take advantage of this secretion system with the aim of obtaining, for instance, an efficient and smart drug delivery system, once the molecular mechanisms involved in cargo selection and vesicle packaging are uncovered. In fact, MVs could be even used for the delivery of specific cargo to target cells. Indeed, these vesicles are expected to have, in nature, the ability to fuse with other membranous structures.

## MATERIALS AND METHODS

### Bacterial strain and culturing conditions.

Streptomyces coelicolor A3 (2) M145 SCP1- SCP2- (67) was used. S. coelicolor seed culture was prepared by inoculating an amount of ∼10^8^ spores in 30 mL of J Medium (67) for 30 h at 30°C under shaking at 200 rpm. Biomass was harvested by centrifugation at 3000 × *g* for 10 min at 4°C; after two washes with sterile water, it was resuspended in 30 mL of sterile water. Then, 10 mL of the bacterial suspension was inoculated in 500 mL of minimal medium (MM) (32) and incubated at 30°C for 136 h, under shaking at 180 rpm.

### Scanning electron microscopy observations.

Fifty-μL aliquots from six-day-old liquid culture of S. coelicolor was placed on coverslips, incubated at room temperature for 30 min, and then fixed using 4% vol/vol glutaraldehyde for 5 min. After the removal of glutaraldehyde, samples were washed twice with 15% vol/vol ethanol and 50% vol/vol ethanol solutions, and incubated at 65°C for 15 min.

The morphology of samples was analyzed by SEM using a Quanta 200 ESEM instrument (FEI, Hillsboro, OR, USA). The samples were attached to an aluminum stub using adhesive carbon tape. All the samples were sputter coated with a thin layer of gold under an argon atmosphere for 90 sec (Scancoat Six Edwards, Crawley, United Kingdom) to avoid electrostatic charging under the electron beam. SEM micrographs were taken in secondary electron imaging mode using an accelerating voltage of 15.0 kV, spot size 3.5 at a working distance of about 10 mm.

### Isolation and purification of membrane vesicles.

MVs were isolated and purified according to Prados-Rosales et al. 2014 (21), with minor modifications. In brief, the culture supernatant was prepared by centrifuging the bacterial culture at 4,000 × *g* for 10 min at 4°C, and then it was filtered through a 0.2-μm pore size filter. The filtrate was concentrated using an Amicon Ultrafiltration system with a 100-kDa exclusion filter. The concentrated supernatant was subjected to sequential centrifugations at 4,000 and 15,000 × *g* for 15 min at 4°C. The remaining supernatant was ultracentrifugated at 100,000 × *g* (SW 40 Ti Rotor, Beckman Coulter) for 1 h at 4°C. The MV pellet was suspended in Dulbecco's phosphate-buffered saline (DPBS) without Ca^2+^ and Mg^2+^ ions (Gibco), and mixed with the OptiPrep solution (Sigma-Aldrich) to obtain a final 35% vol/vol OptiPrep solution. The sample was loaded into a 14-mL ultracentrifugation tube and 2 mL of OptiPrep solutions with decreasing concentration (from lower to upper: 30%, 25%, 20%, 15%, 10% vol/vol) were layered on top. Following this, samples were ultracentrifugated at 140,000 × *g* for 16 h at 4°C, using a swinging rotor (SW 40 Ti Rotor, Beckman Coulter). Next, all six gradient fractions (F1 through F6) were collected proceeding from the top, diluted with DPBS and finally ultracentrifugated at 38,400 × *g* (SW 55 Ti Rotor, Beckman Coulter) for 2 h at 4°C. Pellets were suspended in 0.2 mL of sterile DPBS and aliquots were streaked on Soya Flour Mannitol medium (SFM) (67) and MM plates to verify their sterility; they were stored at –80°C until use. Fractions were examined by 12% T SDS-PAGE and silver staining (68) for evaluation of the corresponding protein content.

### Transmission electron microscopy observations.

Twenty-μL aliquots of MVs from fractions F3 and F4 were pretreated for 15 min with 0.25% vol/vol glutaraldehyde. Samples were placed onto carbon-covered Cu grids (300 mesh, Plano). After removal of excess liquid, neutralized 3% wt/vol phosphotungstic acid was added for 1 min. Grids were then rinsed with drops of distilled water (69). Air-dried samples were analyzed by TEM (JEOL Japan JEM-2100) operating at 120 and 200 kV.

### Atomic force microscopy observations.

Twenty-μL aliquots of MVs from fractions F3 and F4 (diluted 1:10 in H_2_O) were deposited on a mica substrate, and then dried under vacuum (10 mbar). AFM measurements were carried out in soft tapping mode in air using a Bruker FAST-SCAN microscope. The probes used were FastScan A, with a nominal tip radius of 5 nm, spring constant of 18 N/m, and resonance frequency of 1,400 KHz. The scanner was calibrated using a 1 μm × 1 μm reference grid. AFM images were obtained with a tip velocity of 20 μm/s and a target amplitude of ∼15 nm. The pixel resolution was fixed at about 1,000 × 1,000 points. Each sample was typically characterized by acquiring five images obtained at different points.

### Dynamic light scattering measurements.

The size distribution of MVs was determined with DLS using a Malvern Zetasizer NanoZS instrument, fitted with a 532 nm laser at a fixed scattering angle of 173°. The intensity-average hydrodynamic diameter and PDI were obtained by cumulant analysis of the correlation function. Aliquots of 100-μL MVs (diluted 1:10 in H_2_O) were loaded into disposable sizing cuvettes at 25°C, and the system was assigned with a dispersant refractive index of 1.330 and a viscosity value of 0.8872. The average diameter (Z-average) and PDI of MVs were determined with Zetasizer software.

### Vesicle shaving experiments.

Forty-μL aliquots of MVs from fractions F3 and F4 were either treated or not treated with 0.5 μL of a solution containing proteinase K (PK) from Tritirachium album (10 μg/mL, [pH 7.1]; Sigma-Aldrich) for 1 h at room temperature. The proteinase activity was then inhibited by adding 0.1 mM phenylmethylsulfonyl fluoride (70). Samples were analyzed in parallel with SDS-PAGE followed by proteomic analyses.

### Proteomic analysis.

Two parallel cultures (biological replicates) were performed to obtain MVs from fractions F3 and F4, which (in parallel) were either treated or not treated with PK (see above). These samples were subjected to 12% T SDS-PAGE. Forty-μL aliquots were used for each sample and protein patterns were revealed by colloidal Coomassie blue staining. Each lane was cut in 16 slices, which were thoroughly triturated, in-gel reduced with dithiothreitol, S-alkylated with iodoacetamide, and finally digested with trypsin. Peptides were extracted from gel particles using 5% vol/vol formic acid/acetonitrile (1:1 vol/vol), and digest solutions were concentrated and desalted before mass spectrometry analysis using μZip TipC18 pipette tips (Millipore). Peptide mixtures from each sample were analyzed in technical triplicate using a nanoLC-ESI-Q-Orbitrap-MS/MS platform, consisting of a Q ExactivePlus Orbitrap mass spectrometer equipped with a Nanospray Flex ion source (Thermo Fisher Scientific, USA) connected to an UltiMate 3000 RSLC nano-liquid chromatographer (Dionex, USA). Protein digests were separated on a 15 cm (length) × 75 mm (inner diameter) column packed with Acclaim PepMap RSLC C18 resin (Thermo Fisher Scientific). Mobile phases were 0.1% vol/vol formic acid in water (eluent A) and 0.1% vol/vol formic acid in acetonitrile/water 4/1 vol/vol (eluent B), running at a total flow rate of 300 nL/min. A linear gradient started 20 min after sample loading; eluent B ramped from 3% to 40% vol/vol over 40 min, and from 40% to 80% vol/vol over 5 min. The mass spectrometer operated in data-dependent scan mode, allowing the acquisition of all MS spectra in the positive ionization mode within a scan range of 375 to 1500 m/z. Up to 8 of the most intense precursor ions in MS were selected for higher energy collisional ion fragmentation. A nominal resolving power of 70,000 full-width at half-maximum (FWHM), an automatic gain control (AGC) target of 3 × 10^6^ ions, and a maximum ion injection time (IT) of 80 ms were set to generate precursor spectra. MS/MS fragmentation spectra were obtained by applying a normalized collision energy of 28%, a nominal resolving power of 17,500 FWHM, an AGC target of 5 × 10^4^ ions, a maximum IT of 110 ms, and an isolation window of 1.2 m/z. To prevent repeated fragmentation of the most abundant precursor ions, a dynamic exclusion of 20 s was applied. Singly charged precursor ions or those with more than six charges were excluded from the fragmentation. The MS/MS raw data were subjected to protein database searches using Proteome Discoverer Software 2.1 (Thermo Fisher Scientific, USA), enabling database search by Mascot engine v. 2.4.2 (Matrix Science, UK) using the following criteria. UniProtKB protein database for S. coelicolor (downloaded in April 2018), including the most common protein contaminants plus PK. Carbamidomethylation of Cys as static modification and oxidation of Met as variable modification were used. Precursor ions mass tolerance was fixed to ±10 ppm, and fragment mass tolerance to ±0.05 Da. The maximum number of missed cleavages was set to 2. All other parameters were kept as default. Protein candidates were considered identified when at least two peptides were sequenced and the corresponding protein false discovery rate (FDR) confidence was “high”. According to software output, the latter corresponds to an FDR value of 0.01 (1% of total probability 100%). Identification details of all proteins are reported in Table S2. Proteins that were detected as common to all technical replicates run for each biological sample are reported in the latter table. Regarding the variability between biological replicates of the same sample type, proteomic analyses always showed common entries between replicates I and II, which accounted for about 50 to 60% of the total proteins identified therein. To avoid missing important information, all identified proteins (whether or not they were common to replicates I and II) were considered for further bioinformatics analyses.

Since large proteins after proteolytic digestion tend to generate more peptides/mass spectra than small ones, the normalized spectral abundance factor (NSAF) constitutes a useful tool for evaluating the effect of protein length on spectral count. Indeed, NSAF is defined as the number of spectral counts (SpC) identifying a protein divided by that protein’s length (L), divided by the sum of SpC/L for all proteins in the experiment. Hence, NSAF values enable comparison of the abundance of individual proteins across multiple independent samples (71). The ratio between NSAF values of untreated MVs and PK-treated MVs for the same protein constituted a strong indication for protein localization in the vesicles. In particular, proteins with an NSAF ratio of >2 were categorized as associated with vesicle surface (out); when the value was <1.5, proteins were categorized as internal (in); and proteins with NSAF ratios between 1.5 and 2 were not reliably assigned to a localization (unknown) (Table S1).

The results of MS identification were used to perform a gene ontology study. Metabolic pathways were reconstructed using the protein KEGG database (Kyoto Encyclopedia of Genes and Genomes). Predictions of protein localization and signal peptides were carried out by SToPSdb v.1 beta (http://stopsdb.eu/step2golist.php) and SignalP 5.0 (https://services.healthtech.dtu.dk/service.php?SignalP-5.0), respectively.

### Targeted metabolomic analysis.

After protein precipitation and the extraction of metabolites in organic solvents, the metabolomic profile of vesicle samples was carried out by LC-MS/MS analysis with a mass spectrometer operating in Multiple Reaction Monitoring (MRM) mode. In particular, 100-mg aliquots of MVs from fractions F3 and F4 (two biological replicates for each) were lysed, adding 250 μL of 10 mM NH_4_CO_3_ and 10 mM NaF buffer containing 7 M urea and 75 mM NaCl. The suspensions were then homogenized in 6 M guanidine buffer using the IKA T-10 Basic Ultra Turrax Homogenizer (IKA-Werke GmbH & Co., Staufen, Germany) for 2 min. Samples were then centrifuged at 12,000 rpm for 10 min. The lysates were diluted 6-fold with cold methanol. The mixtures were sonicated for 10 min and centrifuged at 12,000 rpm for 10 min at 4°C. The volume of the supernatant was reduced to 200 μL. After centrifugation at 12,000 rpm for 10 min, the supernatant was directly analyzed with an Eksigent HPLC system linked on-line to a 4000 QTRAP hybrid triple quadrupole mass spectrometer (AB Sciex, Framingham, MA, USA). Metabolite mixtures (6 μL) were resolved using a Halo C18 column (1 × 50 mm, 2.7-μm particle size, 90-Å pores) at 38°C. Eluents A and B were 0.1% vol/vol acetic acid and acetonitrile/2-propanol/acetic acid 49.95/49.95/0.10 vol/vol/vol, respectively. The elution gradient was from 0% to 95% B in 7 min, at a flow rate of 40 μL/min. Analysis was carried out in MRM mode, with an ESI ionization source. Precursor ion was scanned in negative mode for organic acids, monosaccharides, and other molecules; amino acids, vitamins, and antibiotics were analyzed in positive mode. Precursor (Q1) and daughter ion (Q2), collision energy (CE), and declustering potential (DP) parameters for all molecules monitored in MRM mode were optimized. Quantitative analysis was performed with an external calibration. A solution of standard metabolites was prepared by dissolving 1 mg of each analyte in 1 mL of 5% vol/vol acetonitrile (1,000 ppm), whereas the stock solution of undecylprodigiosin was dissolved in 50% vol/vol acetonitrile. Stock solutions were stored at –20°C until analysis. Serial dilutions were prepared from stock solutions (1,000 ppm) up to 0.01 ppb, and used to generate calibration curves. The amount of metabolites in each fraction was determined in ppm, and averaged using results from duplicated analyses for each sample. After this, the fold change for each metabolite was calculated by normalizing the result of the F3 fraction to that of the F4 counterpart. Experiments were carried out at the Centro di Ingegneria Genetica (CEINGE) Biotecnologie Avanzate, Naples, Italy.

### Untargeted metabolomic analysis.

After protein precipitation and extraction of metabolites in organic solvents, untargeted metabolomics of vesicle samples was carried by LC-MS/MS analysis. Vesicle samples were analyzed with an Agilent 1260 HPLC system linked on-line to an Agilent 6540 UHD accurate-mass quadrupole time-of-flight (Q-TOF) spectrometer (Palo Alto, CA, USA) equipped with a Dual AJS ESI source working in either positive or negative mode; separate runs for each polarity were performed. Metabolite mixtures were resolved using a ZORBAX Extended-C18 reversed-phase column (2.1 × 50 mm, 1.8-μm particle size) equipped with a Phenomenex C18 security guard column (4 × 3 mm), at 30°C. Eluents A and B were 0.1% vol/vol formic acid and acetonitrile/formic acid 99.9/0.1 vol/vol, respectively. Elution gradient was from 5% to 95% B in 10 min, followed by an isocratic step at 95% B for column washing and reconditioning, at a flow rate of 0.4 mL/min. The eluate was monitored through MS total ion current (TIC). N_2_ was used as a desolvation gas at 300°C and with a flow rate of 9 L/min. The nebulizer was set to 45 lb/in^2^. The sheath gas temperature was set at 350°C and a flow of 12 L/min. Potentials of 3.5 and 2.6 kV were used on the capillary for positive and negative ion mode, respectively. The fragmentor was set to 175 V. MS spectra were recorded in the 50 to 1,500 m/z range. Molecule identification was performed by comparison with the Metlin database (Scripps Center for Metabolomics, https://metlin.scripps.edu) or with available standard compounds.

### Raman spectroscopy analysis.

Five-μL aliquots of MV fractions F3 and F4 were placed on glass slides and dried for Raman spectroscopy analysis, which was performed on a Horiba HR-Evolution confocal Micro-Raman spectrometer equipped with a 633 nm laser line and a 600 l/mm grating, with a spectral resolution of 6 cm^−1^. Each pixel has been acquired by a maximum of 30 laser repetitions. For all of the measurements, a minimum laser power of <8 mW was applied to avoid sample degradation, and the long working distance (NA 0.50) objective with ×50 magnification was typically used to maximize the spatial resolution. Actinorhodin was used as a standard, as resulting from an extraction with 1 N KOH (67).

### Data availability.

Proteomics data have been deposited to the ProteomeXchange Consortium via the PRIDE (72) partner repository with the data set identifier PXD024085.
