# Supplementary material for: Streptomyces coelicolor Vesicles: Many Molecules To Be Delivered
Source: Appl Environ Microbiol. 2022 Jan 11;88(1):e01881-21. doi: 10.1128/AEM.01881-21 (PMC8752130; doi:10.1128/AEM.01881-21)
Supplement: Supplemental file 1 — Tables S1, S3, and S4, Fig. S1 to S3. Download AEM.01881-21-s0001.pdf, PDF file, 1.9 MB [file aem.01881-21-s0001.pdf]

Streptomyces coelicolor vesicles: many molecules to be delivered

Teresa Faddetta, [a#] Giovanni Renzone, [b]Alberto Vassallo, [c] Emilio Rimini, [a] Giorgio Nasillo,[d] Gianpiero Buscarino,[e] Simonpietro Agnello,[e] Mariano Licciardi,[a] Luigi Botta,[f] Andrea Scaloni,[b] Antonio Palumbo Piccionello,[a] Anna Maria Puglia[a] and Giuseppe Gallo[a]

[a]Department of Biological Chemical and Pharmaceutical Sciences and Technology (STEBICEF), University of Palermo, Palermo, Italy

[b]Proteomic and Mass Spectrometry Laboratory, ISPAAM, Consiglio Nazionale delle Ricerche Napoli, Italy

[c]Department of Biology, University of Florence, Sesto Fiorentino, Italy

[d]Advanced Technologies Network (ATeN Center), University of Palermo, Palermo, Italy

[e]Department of Physics and Chemistry, University of Palermo, Palermo, Italy

[f]Department of Engineering, University of Palermo, Palermo, Italy

Running Head: Membrane vesicles of *Streptomyces coelicolor*

# Address correspondence to Teresa Faddetta, teresa.faddetta@unipa.it

SUPPLEMETAL MATERIAL

Table S1: Proteins identified in *S. coelicolor* MVs. For each protein, reported are UniProtKB accession, description, assignment at the luminal and/or external portion of MVs, SCO accession, gene name, functional classification and subcellular localization.

	UniProtKB accession number	Description	F3	F4	SCO	Gene	KEGG Orthology	Subcellular localization
1	P12690	Spore-associated protein A	IN	IN	SCO0409	sapA	Unknown	Extracellular
2	Q9RJM5	NH(3)-dependent NAD(+) synthetase	-	OUT	SCO0506	nadE	•Metabolism of cofactors and vitamins<br>Nicotinate and nicotinamide metabolism	Peripheral inner membrane protein facing the cytoplasm
3	P48859	Cold shock protein ScoF	OUT	-	SCO0527	scoF	•Unclassified: genetic information processing<br>Transcription	Cytoplasmic
4	Q9RJH9	Catalase-peroxidase	OUT	OUT	SCO0560	katG	•Amino acid metabolism<br>Phenylalanine metabolism<br>Tryptophan metabolism	Cytoplasmic
5	Q9XA86	Putative membrane protein SCO0839	OUT	-	SCO0839	/	Unknown	Integral membrane
6	Q93J59	5-methyltetrahydropteroyltriglutamate--homocysteine methyltransferase	OUT	IN	SCO0985	metE	•Amino acid metabolism<br>Cysteine and methionine metabolism<br>•Metabolism of other amino acids<br>Selenocompound metabolism	Cytoplasmic
7	Q9FCD7	Tripeptidyl aminopeptidase	UNKNOWN	IN	SCO1230	tap	Unknown	Extracellular; Vesicles
8	Q8CK28	ATP phosphoribosyltransferase	-	OUT	SCO1438	hisG	•Amino acid metabolism<br>Histidine metabolism	Cytoplasmic
9	Q9EWJ9	6,7-dimethyl-8-ribityllumazine synthase	OUT	UNKNOWN	SCO1440	ribH	•Metabolism of cofactors and vitamins<br>Riboflavin metabolism	Peripheral inner membrane protein facing the cytoplasm

10	Q9L0Y3	S-adenosylmethionine synthase	OUT	OUT	SCO1476	metK	•Amino acid metabolism<br>Cysteine and methionine metabolism	Peripheral inner membrane protein facing the cytoplasm
11	Q9KXR7	Dihydroorotate dehydrogenase (quinone)	IN	-	SCO1482	pyrD	•Nucleotide metabolism<br>Pyrimidine metabolism	Peripheral inner membrane protein facing the cytoplasm
12	Q9KXP5	30S ribosomal protein S4	OUT	UNKNOWN	SCO1505	rpsD	•Translation<br>Ribosome<br>•Protein families: genetic information processing<br>Ribosome	Ribosomal
13	Q53956	Protein translocase subunit SecF	IN	IN	SCO1515	secF	•Folding, sorting and degradation<br>Protein export<br>•Membrane transport<br>Bacterial secretion system<br>•Protein families: signaling and cellular processes<br>Secretion system	Integral membrane
14	Q53955	Protein translocase subunit SecD	OUT	-	SCO1516	secD	•Folding, sorting and degradation<br>Protein export<br>•Membrane transport<br>Bacterial secretion system<br>•Protein families: signaling and cellular processes<br>Secretion system	Integral membrane
15	Q9L278	Threonine--tRNA ligase	OUT	OUT	SCO1531	thrS	•Translation<br>Aminoacyl-tRNA biosynthesis<br>•Protein families: metabolism<br>Amino acid related enzymes<br>•Protein families: genetic information processing<br>Transfer RNA biogenesis	Peripheral inner membrane protein facing the cytoplasm

16	O88054	Phenylalanine--tRNA ligase beta subunit	OUT	-	SCO1594	pheT	•Translation<br>Aminoacyl-tRNA biosynthesis<br>•Protein families: metabolism<br>Amino acid related enzymes<br>•Protein families: genetic information processing<br>Transfer RNA biogenesis	Peripheral inner membrane protein facing the cytoplasm
17	O88058	50S ribosomal protein L20	IN	UNKNOWN	SCO1598	rplT	•Translation<br>Ribosome<br>•Protein families: genetic information processing<br>Ribosome	Ribosomal
18	O88059	50S ribosomal protein L35	OUT	-	SCO1599	rpmI	•Translation<br>Ribosome<br>•Protein families: genetic information processing<br>Ribosome	Ribosomal
19	O88060	Translation initiation factor IF-3	OUT	UNKNOWN	SCO1600	infC	•Protein families: genetic information processing<br>Translation factors	Peripheral inner membrane protein facing the cytoplasm
20	Q9RJ68	Sec-independent protein translocase protein TatA	IN	-	SCO1633	tatA	•Folding, sorting and degradation<br>Protein export<br>•Membrane transport<br>Bacterial secretion system<br>•Protein families: signaling and cellular processes<br>Secretion system	Cytoplasmic, Integral membrane
21	Q9RJ61	Pup--protein ligase	OUT	UNKNOWN	SCO1640	pafA	•Protein families: genetic information processing<br>Proteasome	Cytoplasmic
22	P19255	Probable glycerol uptake facilitator protein	UNKNOWN	IN	SCO1659	glpF	•Protein families: signaling and cellular processes<br>Transporters	Integral membrane
23	Q9XAC0	Transaldolase 2	OUT	UNKNOWN	SCO1936	tal2	•Carbohydrate metabolism<br>Pentose phosphate pathway	Cytoplasmic

24	Q9Z523	Glucose-6-phosphate isomerase 2	OUT	-	SCO1942	pgi2	•Carbohydrate metabolism<br>Glycolysis / Gluconeogenesis<br>Pentose phosphate pathway<br>Starch and sucrose metabolism<br>Amino sugar and nucleotide sugar metabolism<br>•Protein families: signaling and cellular processes<br>Exosome	Cytoplasmic
25	Q9Z520	Triosephosphate isomerase	IN	-	SCO1945	tpiA	•Carbohydrate metabolism<br>Glycolysis / Gluconeogenesis<br>Fructose and mannose metabolism<br>Inositol phosphate metabolism<br>•Protein families: signaling and cellular processes<br>Exosome	Peripheral inner membrane protein facing the cytoplasm
26	Q9Z519	Phosphoglycerate kinase	IN	-	SCO1946	pgk	•Carbohydrate metabolism<br>Glycolysis / Gluconeogenesis<br>•Protein families: signaling and cellular processes<br>Exosome	Peripheral inner membrane protein facing the cytoplasm
27	Q9Z518	Glyceraldehyde-3-phosphate dehydrogenase	OUT	UNKNOWN	SCO1947	gap	•Carbohydrate metabolism<br>Glycolysis / Gluconeogenesis<br>•Protein families: genetic information processing<br>Membrane trafficking<br>• Protein families: signaling and cellular processes<br>Exosome	Peripheral inner membrane protein facing the cytoplasm
28	Q9Z513	Nucleotide-binding protein SCO1952	-	OUT	SCO1952	/	•Protein families: genetic information processing<br>Messenger RNA biogenesis	Cytoplasmic
29	Q9S2U8	Prolipoprotein diacylglyceryl transferase 1	IN	IN	SCO2034	lgt1	Unknown	Integral membrane

30	Q9S2X7	Lipoprotein signal peptidase	IN	-	SCO2074	lspA	•Folding, sorting and degradation<br>Protein export<br>•Protein families: metabolism<br>Peptidases	Integral membrane
31	Q9S2X5	Isoleucine--tRNA ligase	-	IN	SCO2076	ileS	•Translation<br>Aminoacyl-tRNA biosynthesis<br>•Protein families: metabolism<br>Amino acid related enzymes<br>•Protein families: genetic information processing<br>Transfer RNA biogenesis	Cytoplasmic
32	Q9S2X2	Cell division protein SepF 2	IN	-	SCO2079	sepF2	•Protein families: genetic information processing<br>Chromosome and associated proteins	Peripheral inner membrane protein facing the cytoplasm
33	Q9ZBA5	UDP-N-acetylglucosamine--N-acetylmuramyl-(pentapeptide) pyrophosphoryl-undecaprenol N-acetylglucosamine transferase	OUT	-	SCO2084	murG	•Glycan biosynthesis and metabolism<br>Peptidoglycan biosynthesis<br>•Drug resistance: Antimicrobial<br>Vancomycin resistance<br>•Protein families: metabolism<br>Peptidoglycan biosynthesis and degradation proteins	Peripheral inner membrane protein facing the cytoplasm
34	P56833	Phospho-N-acetylmuramoyl-pentapeptide-transferase	IN	-	SCO2087	mraY	•Glycan biosynthesis and metabolism<br>Peptidoglycan biosynthesis<br>•Drug resistance: Antimicrobial<br>Vancomycin resistance<br>•Protein families: metabolism<br>Peptidoglycan biosynthesis and degradation proteins	Integral membrane
35	Q9S2N0	Bacterioferritin	IN	IN	SCO2113	bfr	•Metabolism of cofactors and vitamins<br>Porphyrin and chlorophyll metabolism	Cytoplasmic

36	P80574	Phospho-2-dehydro-3-deoxyheptonate aldolase	-	OUT	SCO2115	aroH	•Amino acid metabolism<br>Phenylalanine, tyrosine and tryptophan biosynthesis<br>•Cellular community - prokaryotes<br>Quorum sensing	Cytoplasmic
37	Q9X806	Cytochrome bc1 complex cytochrome b subunit	IN	IN	SCO2148	qcrB	•Energy metabolism<br>Oxidative phosphorylation	Integral membrane
38	Q9X807	Cytochrome bc1 complex Rieske iron-sulfur subunit	IN	IN	SCO2149	qcrA	•Energy metabolism<br>Oxidative phosphorylation	Integral membrane
39	Q9X813	Probable cytochrome c oxidase subunit 1-alpha	IN	IN	SCO2155	ctaD1	•Energy metabolism<br>Oxidative phosphorylation	Integral membrane
40	Q9X814	Probable cytochrome c oxidase subunit 2	IN	IN	SCO2156	ctaC/cox	•Energy metabolism<br>Oxidative phosphorylation	Integral membrane
41	Q9S2R1	Nicotinate-nucleotide--dimethylbenzimidazole phosphoribosyltransferase	OUT	-	SCO2175	cobT	•Metabolism of cofactors and vitamins<br>Porphyrin and chlorophyll metabolism	Cytoplasmic
42	Q9S2Q7	Probable cytosol aminopeptidase	OUT	IN	SCO2179	pepA	•Metabolism of other amino acids<br>Glutathione metabolism<br>•Protein families: metabolism<br>Peptidases	Peripheral inner membrane protein facing the cytoplasm
43	P15106	Glutamine synthetase	OUT	OUT	SCO2198	glnA	•Carbohydrate metabolism<br>Glyoxylate and dicarboxylate metabolism<br>•Energy metabolism<br>Nitrogen metabolism<br>•Amino acid metabolism<br>Alanine, aspartate and glutamate metabolism<br>Arginine biosynthesis<br>•Signal transduction<br>Two-component system	Cytoplasmic

44	Q9L2H4	Isoprenyl transferase 1	IN	-	SCO2509	uppS1	•Metabolism of terpenoids and polyketides<br>Terpenoid backbone biosynthesis<br>• Protein families: metabolism<br>Prenyltransferases	Cytoplasmic
45	Q9RDE2	Tricorn protease homolog 1	OUT	-	SCO2549	tri1	•Protein families: metabolism<br>Peptidases	Cytoplasmic
46	Q9RDM3	30S ribosomal protein S20	OUT	-	SCO2563	rpsT	•Translation<br>Ribosome<br>•Protein families: genetic information processing<br>Ribosome	Ribosomal
47	Q9L1I1	50S ribosomal protein L27	OUT	OUT	SCO2596	rpmA	•Translation<br>Ribosome<br>•Protein families: genetic information processing<br>Ribosome	Ribosomal
48	Q9L1I0	50S ribosomal protein L21	OUT	-	SCO2597	rplU	•Translation<br>Ribosome<br>•Protein families: genetic information processing<br>Ribosome	Ribosomal
49	O06851	Valine--tRNA ligase	-	OUT	SCO2615	valS	•Translation<br>Aminoacyl-tRNA biosynthesis<br>•Protein families: metabolism<br>Amino acid related enzymes<br>•Protein families: genetic information processing<br>Transfer RNA biogenesis	Cytoplasmic
50	Q9ZH58	ATP-dependent Clp protease proteolytic subunit 2	OUT	-	SCO2618	clpP2	•Protein families: metabolism<br>Peptidases	Cytoplasmic
51	Q9F315	ATP-dependent Clp protease proteolytic subunit 1	-	OUT	SCO2619	clpP1	•Protein families: metabolism<br>Peptidases	Cytoplasmic
52	O51917	Superoxide dismutase [Fe-Zn] 1	IN	IN	SCO2633	sodF1	Unknown	Cytoplasmic

53	Q9RDI1	Ribose import ATP-binding protein RbsA 2	OUT	-	SCO2746	rbsA2	•Membrane transport<br>ABC transporters<br>•Protein families: signaling and cellular processes<br>Transporters	Peripheral inner membrane protein facing the cytoplasm
54	P54740	Serine/threonine-protein kinase PkaB	OUT	-	SCO2973	pkaB	•Protein families: metabolism<br>Protein kinases	Integral membrane
55	P54739	Serine/threonine-protein kinase PkaA	IN	-	SCO2974	pkaA	•Protein families: metabolism<br>Protein kinases	Integral membrane
56	P0A4G6	Protein translocase subunit SecA	OUT	OUT	SCO3005	secA	•Folding, sorting and degradation<br>Protein export<br>•Membrane transport<br>Bacterial secretion system<br>•Cellular community - prokaryotes<br>Quorum sensing<br>•Protein families: signaling and cellular processes<br>Secretion system	Peripheral inner membrane protein facing the cytoplasm
57	Q9KZM1	Adenosylhomocysteinase	-	OUT	SCO3023	ahcY	•Amino acid metabolism<br>Cysteine and methionine metabolism<br>•Protein families: metabolism<br>Protein phosphatases and associated proteins<br>•Protein families: signaling and cellular processes<br>Exosome	Cytoplasmic

58	Q9F2Q3	Enolase 1	OUT	OUT	SCO3096	eno1	•Carbohydrate metabolism<br>Glycolysis / Gluconeogenesis<br>•Energy metabolism<br>Methane metabolism<br>•Folding, sorting and degradation<br>RNA degradation<br>•Protein families: genetic information processing<br>Messenger RNA biogenesis<br>•Protein families: signaling and cellular processes<br>Exosome	Cytoplasmic
59	Q9K3U0	Ribose-phosphate pyrophosphokinase	-	OUT	SCO3123	prs	•Carbohydrate metabolism<br>Pentose phosphate pathway<br>•Nucleotide metabolism<br>Purine metabolism	Peripheral inner membrane protein facing the cytoplasm
60	Q9RNU9	Phosphoenolpyruvate carboxylase	UNKNOWN	UNKNOWN	SCO3127	ppc	•Carbohydrate metabolism<br>Pyruvate metabolism<br>•Energy metabolism<br>Methane metabolism	Peripheral inner membrane protein facing the cytoplasm
61	Q9RKA8	Cyclic pyranopterin monophosphate synthase	-	IN	SCO3180	moaC	•Metabolism of cofactors and vitamins<br>Folate biosynthesis<br>•Folding, sorting and degradation<br>Sulfur relay system	Cytoplasmic
62	Q9X895	Lysine--tRNA ligase	-	OUT	SCO3303	lysS	•Translation<br>Aminoacyl-tRNA biosynthesis<br>•Protein families: metabolism<br>Amino acid related enzymes<br>•Protein families: genetic information processing<br>Transfer RNA biogenesis	Cytoplasmic

63	Q9WX29	Arginine--tRNA ligase	OUT	UNKNOWN	SCO3304	argS	•Translation<br>Aminoacyl-tRNA biosynthesis<br>•Protein families: metabolism<br>Amino acid related enzymes<br>•Protein families: genetic information processing<br>Transfer RNA biogenesis	Cytoplasmic
64	Q9X8L6	DNA integrity scanning protein DisA	OUT	OUT	SCO3352	disA	Unknown	Cytoplasmic
65	Q9ZEP4	Transcriptional regulatory protein CseB	-	OUT	SCO3358	cseB	Unknown	Nucleoid
66	Q9X913	K(+)-insensitive pyrophosphate-energized proton pump	IN	IN	SCO3547	hppA	•Protein families: signaling and cellular processes<br>Transporters	Integral membrane
67	Q9WVX8	Anti-sigma-B factor antagonist	IN	OUT	SCO3549	rsbV/bldG	•Protein families: genetic information processing<br>Transcription machinery	Cytoplasmic
68	Q9X928	Acetyl-coenzyme A synthetase	OUT	OUT	SCO3563	acsA	•Carbohydrate metabolism<br>Glycolysis / Gluconeogenesis<br>Pyruvate metabolism<br>Glyoxylate and dicarboxylate metabolism<br>Propanoate metabolism<br>•Energy metabolism<br>Methane metabolism<br>•Protein families: metabolism<br>Lipid biosynthesis proteins	Peripheral inner membrane protein facing the cytoplasm
69	Q9X8R6	Fructose-bisphosphate aldolase	UNKNOWN	-	SCO3649	fba	•Carbohydrate metabolism<br>Glycolysis / Gluconeogenesis<br>Pentose phosphate pathway<br>Fructose and mannose metabolism<br>•Energy metabolism<br>Methane metabolism	Peripheral inner membrane protein facing the cytoplasm

70	Q05558	Chaperone protein DnaK	UNKNOWN	OUT	SCO3671	dnaK	•Folding, sorting and degradation<br>RNA degradation<br>•Protein families: genetic information processing<br>Messenger RNA biogenesis<br>Chaperones and folding catalysts<br>•Protein families: signaling and cellular processes<br>Exosome	Peripheral inner membrane protein facing the cytoplasm
71	Q9F2I9	Methionine--tRNA ligase	OUT	OUT	SCO3792	metG	•Translation<br>Aminoacyl-tRNA biosynthesis<br>•Protein families: metabolism<br>Amino acid related enzymes<br>•Protein families: genetic information processing<br>Transfer RNA biogenesis	Cytoplasmic
72	Q9F323	Aspartate--tRNA ligase	OUT	UNKNOWN	SCO3795	aspS	•Translation<br>Aminoacyl-tRNA biosynthesis<br>•Protein families: metabolism<br>Amino acid related enzymes<br>•Protein families: genetic information processing<br>Transfer RNA biogenesis	Peripheral inner membrane protein facing the cytoplasm
73	Q9S2C0	Serine/threonine-protein kinase PksC	UNKNOWN	-	SCO3821	pksC	•Protein families: metabolism<br>Protein kinases	Integral membrane
74	Q9XA16	Probable serine/threonine-protein kinase SCO3848	IN	IN	SCO3848	/	•Protein families: metabolism<br>Protein kinases	Integral membrane
75	P35885	DNA gyrase subunit A	IN	UNKNOWN	SCO3873	gyrA	•Protein families: genetic information processing<br>DNA replication proteins<br>DNA repair and recombination proteins	Cytoplasmic

76	P35886	DNA gyrase subunit B	IN	OUT	SCO3874	gyrB	•Protein families: genetic information processing<br>DNA replication proteins<br>DNA repair and recombination proteins	Peripheral inner membrane protein facing the cytoplasm
77	P27903	Beta sliding clamp	OUT	OUT	SCO3878	dnaN	•Replication and repair<br>DNA replication<br>Mismatch repair<br>Homologous recombination<br>•Protein families: genetic information processing<br>DNA replication proteins<br>DNA repair and recombination proteins	Cytoplasmic
78	O54569	Membrane protein insertase YidC	IN	IN	SCO3883	yidC	•Folding, sorting and degradation<br>Protein export<br>•Membrane transport<br>Bacterial secretion system<br>•Cellular community - prokaryotes<br>Quorum sensing	Integral membrane
79	Q9X8U3	Single-stranded DNA-binding protein 2	IN	IN	SCO3907	ssb2	•Replication and repair<br>DNA replication<br>Mismatch repair<br>Homologous recombination<br>•Protein families: genetic information processing<br>DNA replication proteins<br>DNA repair and recombination proteins	Nucleoid
80	P66470	30S ribosomal protein S18 1	UNKNOWN	-	SCO3908	rpsR1	•Translation<br>Ribosome<br>•Protein families: genetic information processing<br>Ribosome	Ribosomal
81	Q53868	Uncharacterized protein SCO3922	IN	-	SCO3922	/	Unknown	Integral membrane

82	Q53867	Uncharacterized protein SCO3923	OUT	-	SCO3923	/	Unknown	Cytoplasmic
83	Q9ZBY8	Putative phenylalanine aminotransferase	-	OUT	SCO3944	pat/hisC2	•Amino acid metabolism<br>Histidine metabolism<br>Tyrosine metabolism<br>Phenylalanine metabolism<br>Phenylalanine, tyrosine and tryptophan biosynthesis<br>•Biosynthesis of other secondary metabolites<br>Novobiocin biosynthesis<br>•Protein families: metabolism<br>Amino acid related enzymes	Cytoplasmic
84	Q9ZBX1	Serine--tRNA ligase	-	OUT	SCO3961	serS	•Translation<br>Aminoacyl-tRNA biosynthesis<br>•Protein families: metabolism<br>Amino acid related enzymes<br>•Protein families: genetic information processing<br>Transfer RNA biogenesis	Integral membrane
85	Q9KZV0	Mycothiol acetyltransferase	IN	-	SCO4151	mshD	Unknown	Cytoplasmic
86	P33158	2,3-bisphosphoglycerate-dependent phosphoglycerate mutase	OUT	OUT	SCO4209	gpmA	•Carbohydrate metabolism<br>Glycolysis / Gluconeogenesis<br>•Energy metabolism<br>Methane metabolism<br>•Amino acid metabolism<br>Glycine, serine and threonine metabolism<br>•Protein families: genetic information processing<br>Membrane trafficking<br>•Protein families: signaling and cellular processes<br>Exosome	Peripheral inner membrane protein facing the cytoplasm

87	Q9KXU5	60 kDa chaperonin 2	OUT	OUT	SCO4296	groL2	•Folding, sorting and degradation<br>RNA degradation<br>•Protein families: genetic information processing<br>Messenger RNA biogenesis<br>Chaperones and folding catalysts<br>•Protein families: signaling and cellular processes<br>Exosome	Peripheral inner membrane protein facing the cytoplasm
88	Q9XAQ7	NADH-quinone oxidoreductase subunit D 2	OUT	IN	SCO4565	nuoD2	•Energy metabolism<br>Oxidative phosphorylation	Peripheral inner membrane protein facing the cytoplasm
89	Q9XAQ9	NADH-quinone oxidoreductase subunit F	IN	OUT	SCO4567	nuoF	•Energy metabolism<br>Oxidative phosphorylation	Peripheral inner membrane protein facing the cytoplasm
90	Q9XAR0	NADH-quinone oxidoreductase subunit G	UNKNOWN	-	SCO4568	nuoG	•Energy metabolism<br>Oxidative phosphorylation	Peripheral inner membrane protein facing the cytoplasm
91	Q9XAR1	NADH-quinone oxidoreductase subunit H	IN	-	SCO4569	nuoH	•Energy metabolism<br>Oxidative phosphorylation	Integral membrane
92	Q9XAR5	NADH-quinone oxidoreductase subunit L	IN	IN	SCO4573	nuoL	•Energy metabolism<br>Oxidative phosphorylation	Integral membrane
93	Q9F2V2	Protease HtpX homolog 2	UNKNOWN	-	SCO4609	htpX2	•Protein families: metabolism<br>Peptidases	Integral membrane
94	Q9F2U7	UPF0234 protein SCO4614	IN	-	SCO4614	/	•Unclassified: signaling and cellular processes<br>Signaling proteins	Nucleoid

95	P0A4G8	Protein translocase subunit SecE	OUT	IN	SCO4646	secE	•Folding, sorting and degradation<br>Protein export<br>•Membrane transport<br>Bacterial secretion system<br>•Cellular community - prokaryotes<br>Quorum sensing<br>•Protein families: signaling and cellular processes<br>Secretion system	Integral membrane
96	P48950	50S ribosomal protein L1	-	OUT	SCO4649	rplA	•Translation<br>Ribosome<br>•Protein families: genetic information processing<br>Ribosome	Ribosomal
97	P41109	Putative lipoprotein SCO4651	OUT	-	SCO4651	/	Unknown	Secreted Lipoprotein
98	Q9L0L0	DNA-directed RNA polymerase subunit beta	OUT	OUT	SCO4654	rpoB	•Transcription<br>RNA polymerase<br>•Protein families: genetic information processing<br>Transcription machinery<br>DNA repair and recombination proteins	Peripheral inner membrane protein facing the cytoplasm
99	Q8CJT1	DNA-directed RNA polymerase subunit beta'	OUT	UNKNOWN	SCO4655	rpoC	•Transcription<br>RNA polymerase<br>•Protein families: genetic information processing<br>Transcription machinery<br>DNA repair and recombination proteins	Cytoplasmic
100	P0A4A3	30S ribosomal protein S12	UNKNOWN	OUT	SCO4659	rpsL	•Translation<br>Ribosome<br>•Protein families: genetic information processing<br>Ribosome	Ribosomal

101	Q9L0K4	30S ribosomal protein S7	UNKNOWN	UNKNOWN	SCO4660	rspG	•Translation<br>Ribosome<br>•Protein families: genetic information processing<br>Ribosome	Ribosomal
102	P40173	Elongation factor G 1	-	OUT	SCO4661	fusA	•Protein families: genetic information processing<br>Translation factors	Peripheral inner membrane protein facing the cytoplasm
103	P40174	Elongation factor Tu-1	UNKNOWN	UNKNOWN	SCO4662	tuf1	•Protein families: genetic information processing<br>Translation factors<br>•Protein families: signaling and cellular processes<br>Exosome	Peripheral inner membrane protein facing the cytoplasm
104	P66337	30S ribosomal protein S10	OUT	OUT	SCO4701	rpsJ	•Translation<br>Ribosome<br>•Protein families: genetic information processing<br>Ribosome	Ribosomal
105	Q9L0E0	50S ribosomal protein L3	OUT	OUT	SCO4702	rplC	•Translation<br>Ribosome<br>•Protein families: genetic information processing<br>Ribosome	Ribosomal
106	Q9L0D9	50S ribosomal protein L4	OUT	OUT	SCO4703	rplD	•Translation<br>Ribosome<br>•Protein families: genetic information processing<br>Ribosome	Ribosomal
107	Q9L0D7	50S ribosomal protein L2	OUT	OUT	SCO4705	rplB	•Translation<br>Ribosome<br>•Protein families: genetic information processing<br>Ribosome	Ribosomal

108	Q9L0D6	30S ribosomal protein S19	UNKNOWN	OUT	SCO4706	rpsS	•Translation<br>Ribosome<br>•Protein families: genetic information processing<br>Ribosome	Ribosomal
109	Q9L0D5	50S ribosomal protein L22	OUT	OUT	SCO4707	rplV	•Translation<br>Ribosome<br>•Protein families: genetic information processing<br>Ribosome	Ribosomal
110	Q9L0D4	30S ribosomal protein S3	UNKNOWN	UNKNOWN	SCO4708	rpsC	•Translation<br>Ribosome<br>•Protein families: genetic information processing<br>Ribosome	Ribosomal
111	Q9L0D3	50S ribosomal protein L16	IN	OUT	SCO4709	rplP	•Translation<br>Ribosome<br>•Protein families: genetic information processing<br>Ribosome	Ribosomal
112	Q9L0D0	50S ribosomal protein L14	OUT	UNKNOWN	SCO4712	rplN	•Translation<br>Ribosome<br>•Protein families: genetic information processing<br>Ribosome	Ribosomal
113	Q9L0C9	50S ribosomal protein L24	-	UNKNOWN	SCO4713	rplX	•Translation<br>Ribosome<br>•Protein families: genetic information processing<br>Ribosome	Ribosomal
114	Q9L0C8	50S ribosomal protein L5	UNKNOWN	UNKNOWN	SCO4714	rplE	•Translation<br>Ribosome<br>•Protein families: genetic information processing<br>Ribosome	Ribosomal

115	P66417	30S ribosomal protein S14 type Z	IN	IN	SCO4715	rpsZ	•Translation<br>Ribosome<br>•Protein families: genetic information processing<br>Ribosome	Ribosomal
116	P49399	30S ribosomal protein S8	OUT	OUT	SCO4716	rpsH	•Translation<br>Ribosome<br>•Protein families: genetic information processing<br>Ribosome	Ribosomal
117	P46786	50S ribosomal protein L6	OUT	IN	SCO4717	rplF	•Translation<br>Ribosome<br>•Protein families: genetic information processing<br>Ribosome	Ribosomal
118	P46788	50S ribosomal protein L18	OUT	-	SCO4718	rplR	•Translation<br>Ribosome<br>•Protein families: genetic information processing<br>Ribosome	Ribosomal
119	P46790	30S ribosomal protein S5	OUT	UNKNOWN	SCO4719	rpsE	•Translation<br>Ribosome<br>•Protein families: genetic information processing<br>Ribosome	Ribosomal
120	P46787	50S ribosomal protein L15	OUT	OUT	SCO4721	rplO	•Translation<br>Ribosome<br>•Protein families: genetic information processing<br>Ribosome	Ribosomal
121	P46785	Protein translocase subunit SecY	IN	IN	SCO4722	secY	•Folding, sorting and degradation<br>Protein export<br>•Membrane transport<br>Bacterial secretion system<br>•Cellular community - prokaryotes<br>Quorum sensing	Integral membrane

122	P43414	Adenylate kinase	OUT	-	SCO4723	adk	•Nucleotide metabolism<br>Purine metabolism<br>•Metabolism of cofactors and vitamins<br>Thiamine metabolism<br>•Protein families: signaling and cellular processes<br>Exosome	Peripheral inner membrane protein facing the cytoplasm
123	O86773	30S ribosomal protein S13	OUT	OUT	SCO4727	rpsM	•Translation<br>Ribosome<br>•Protein families: genetic information processing<br>Ribosome	Ribosomal
124	P72403	30S ribosomal protein S11	OUT	OUT	SCO4728	rpsK	•Translation<br>Ribosome<br>•Protein families: genetic information processing<br>Ribosome	Ribosomal
125	P60312	DNA-directed RNA polymerase subunit alpha	-	OUT	SCO4729	rpoA	•Transcription<br>RNA polymerase<br>•Protein families: genetic information processing<br>Transcription machinery<br>DNA repair and recombination proteins	Peripheral inner membrane protein facing the cytoplasm, Nucleoid
126	O86775	50S ribosomal protein L17	UNKNOWN	IN	SCO4730	rplQ	•Translation<br>Ribosome<br>•Protein families: genetic information processing<br>Ribosome	Ribosomal
127	Q53874	50S ribosomal protein L13	UNKNOWN	UNKNOWN	SCO4734	rplM	•Translation<br>Ribosome<br>•Protein families: genetic information processing<br>Ribosome	Ribosomal

128	Q53875	30S ribosomal protein S9	OUT	OUT	SCO4735	rpsI	•Translation<br>Ribosome<br>•Protein families: genetic information processing<br>Ribosome	Ribosomal
129	P40171	60 kDa chaperonin 1	OUT	OUT	SCO4762	groL1	•Folding, sorting and degradation<br>RNA degradation<br>•Protein families: genetic information processing<br>Messenger RNA biogenesis<br>Chaperones and folding catalysts<br>•Protein families: signaling and cellular processes<br>Exosome	Peripheral inner membrane protein facing the cytoplasm
130	Q9KY56	Succinate--CoA ligase [ADP-forming] subunit beta 1	OUT	OUT	SCO4808	sucC1	•Carbohydrate metabolism<br>Citrate cycle (TCA cycle)<br>Propanoate metabolism<br>C5-Branched dibasic acid metabolism	Peripheral inner membrane protein facing the cytoplasm
131	Q9K3J3	Malate dehydrogenase	OUT	OUT	SCO4827	mdh	•Carbohydrate metabolism<br>Citrate cycle (TCA cycle)<br>Pyruvate metabolism<br>Glyoxylate and dicarboxylate metabolism<br>•Energy metabolism<br>Methane metabolism<br>• Amino acid metabolism<br>Cysteine and methionine metabolism	Cytoplasmic

132	Q04943	Signal transduction histidine-protein kinase AfsQ2	OUT	-	SCO4906	afsQ2	•Signal transduction<br>Two-component system<br>•Protein families: metabolism<br>Protein kinases<br>•Protein families: signaling and cellular processes<br>Two-component system	Integral membrane
133	Q04942	Transcriptional regulatory protein AfsQ1	OUT	OUT	SCO4907	afsQ1	Unknown	Nucleoid
134	P46105	Probable actinorhodin transporter	UNKNOWN	IN	SCO5083	actII-2	Unknown	Integral membrane
135	Q53902	Putative membrane protein ActII-3	UNKNOWN	IN	SCO5084	actII-3	Unknown	Integral membrane
136	P16544	Putative ketoacyl reductase	OUT	-	SCO5086	actIII	•Metabolism of terpenoids and polyketides<br>Biosynthesis of type II polyketide backbone<br>Tetracycline biosynthesis<br>•Protein families: metabolism<br>Lipid biosynthesis proteins<br>Polyketide biosynthesis proteins	Cytoplasmic
137	Q9FCI4	UPF0182 protein SCO5204	IN	IN	SCO5204	/	Unknown	Integral membrane
138	Q7AKG9	ECF RNA polymerase sigma factor SigR	IN	-	SCO5216	sigR	•Protein families: genetic information processing<br>Transcription machinery	Cytoplasmic
139	P0A302	ATP synthase protein I	-	IN	SCO5366	atpI	•Protein families: metabolism<br>Photosynthesis proteins	Integral membrane
140	Q9K4D7	ATP synthase subunit b	IN	UNKNOWN	SCO5369	atpF	•Energy metabolism<br>Oxidative phosphorylation	Integral membrane
141	Q9K4D6	ATP synthase subunit delta	IN	-	SCO5370	atpH	•Energy metabolism<br>Oxidative phosphorylation	Peripheral inner membrane protein facing the cytoplasm

142	Q9K4D5	ATP synthase subunit alpha	IN	UNKNOWN	SCO5371	atpA	•Energy metabolism<br>Oxidative phosphorylation	Peripheral inner membrane protein facing the cytoplasm
143	Q9K4D4	ATP synthase gamma chain	IN	-	SCO5372	atpG	•Energy metabolism<br>Oxidative phosphorylation	Peripheral inner membrane protein facing the cytoplasm
144	P0A300	ATP synthase subunit beta	IN	UNKNOWN	SCO5373	atpD	•Energy metabolism<br>Oxidative phosphorylation	Peripheral inner membrane protein facing the cytoplasm
145	P0A2Z6	ATP synthase epsilon chain	IN	-	SCO5374	atpC	•Energy metabolism<br>Oxidative phosphorylation	Peripheral inner membrane protein facing the cytoplasm
146	Q9Z585	DNA ligase 1	OUT	-	SCO5494	ligA1	•Replication and repair<br>DNA replication<br>Base excision repair<br>Nucleotide excision repair<br>Mismatch repair<br>•Protein families: genetic information processing<br>DNA replication proteins<br>DNA repair and recombination proteins	Cytoplasmic
147	Q9ZBR5	50S ribosomal protein L28-1	UNKNOWN	UNKNOWN	SCO5564	rpmB1	•Translation<br>Ribosome<br>•Protein families: genetic information processing<br>Ribosome	Ribosomal
148	Q9ZBR1	Phosphopantetheine adenylyltransferase	-	OUT	SCO5568	coaD	•Metabolism of cofactors and vitamins<br>Pantothenate and CoA biosynthesis	Cytoplasmic

149	Q9ZBQ8	50S ribosomal protein L32-1	OUT	-	SCO5571	rpmF1	•Translation<br>Ribosome<br>•Protein families: genetic information processing<br>Ribosome	Ribosomal
150	O69879	30S ribosomal protein S16	OUT	-	SCO5591	rpsP	•Translation<br>Ribosome<br>•Protein families: genetic information processing<br>Ribosome	Ribosomal
151	P0A477	50S ribosomal protein L19	IN	IN	SCO5595	rplS	•Translation<br>Ribosome<br>•Protein families: genetic information processing<br>Ribosome	Ribosomal
152	O31212	30S ribosomal protein S2	OUT	UNKNOWN	SCO5624	rpsB	•Translation<br>Ribosome<br>•Protein families: genetic information processing<br>Ribosome	Ribosomal
153	O86770	Ribosome-recycling factor	UNKNOWN	-	SCO5627	frr	•Protein families: genetic information processing<br>Translation factors	Peripheral inner membrane protein facing the cytoplasm
154	O86751	Fe(3+) ions import ATP-binding protein FbpC	IN	-	SCO5648	fbpC	•Membrane transport<br>ABC transporters<br>•Protein families: signaling and cellular processes<br>Transporters	Peripheral inner membrane protein facing the cytoplasm
155	Q9KYS0	Putative zinc metalloprotease SCO5695	UNKNOWN	IN	SCO5695	/	Unknown	Integral membrane
156	Q8CJQ8	Translation initiation factor IF-2	OUT	-	SCO5706	infB	•Protein families: genetic information processing<br>Translation factors	Cytoplasmic

157	O86655	30S ribosomal protein S15	IN	IN	SCO5736	rpsO	•Translation<br>Ribosome<br>•Protein families: genetic information processing<br>Ribosome	Ribosomal
158	Q8CJQ6	Polyribonucleotide nucleotidyltransferase	OUT	OUT	SCO5737	pnp	•Folding, sorting and degradation<br>RNA degradation<br>•Protein families: genetic information processing<br>Messenger RNA biogenesis<br>Transfer RNA biogenesis	Cytoplasmic
159	O86841	4-hydroxy-tetrahydrodipicolinate synthase 2	OUT	-	SCO5744	dapA2	•Amino acid metabolism<br>Lysine biosynthesis<br>•Biosynthesis of other secondary metabolites<br>Monobactam biosynthesis	Cytoplasmic
160	O69981	Vitamin B12-dependent ribonucleotide reductase	-	OUT	SCO5805	nrdJ	•Nucleotide metabolism<br>Purine metabolism<br>Pyrimidine metabolism<br>•Protein families: genetic information processing<br>DNA repair and recombination proteins	Cytoplasmic
161	P18183	RNA polymerase principal sigma factor HrdB	IN	-	SCO5820	hrdB	•Protein families: genetic information processing<br>Transcription machinery	Cytoplasmic
162	P0A4I1	Transcriptional regulatory protein CutR	UNKNOWN	IN	SCO5862	cutR	Unknown	Nucleoid
163	P0A4I7	Sensor protein CutS	IN	IN	SCO5863	cutS	Unknown	Integral membrane
164	Q93JF1	Ornithine carbamoyltransferase	-	OUT	SCO5976	argF/arcB	•Amino acid metabolism<br>Arginine biosynthesis	Cytoplasmic

165	Q9FC36	Undecaprenyl-diphosphatase 1	IN	-	SCO7047	uppP1	•Glycan biosynthesis and metabolism<br>Peptidoglycan biosynthesis<br>•Protein families: metabolism<br>Peptidoglycan biosynthesis and degradation proteins	Integral membrane
166	Q9FBT8	Ketol-acid reductoisomerase (NADP(+)) 2	-	OUT	SCO7154	ilvC2	•Amino acid metabolism<br>Valine, leucine and isoleucine biosynthesis<br>•Metabolism of cofactors and vitamins<br>Pantothenate and CoA biosynthesis	Cytoplasmic

Table S3: Results of targeted metabolomics from LC-MS/MS analysis of *S. coelicolor* MVs. Reported are compound name, KEGG ID, functional classification and abundance profile of identified metabolites.

**Metabolites**	**KEGG ID**	**Functional Class**	**F3 MVs (ppm)**	**F4 MVs (ppm)**	**F3 MVs Vs F4 MVs fold change[a]**
Phenylalanine	C00079	amino acids and amino acid precursors	0.049	0.0138	3.6
2-Dehydro-3-Deoxyphosphoheptonate	C04691	amino acids and amino acid precursors	0.12805	0.122	1
6-Phosphogluconate	C04442	components of carbon metabolism	0.0045	0.0046	1
Acetyl Coa	C00024	components of carbon metabolism	0.0085	0.0101	0.8
Actinorhodin	C06691	antibiotics	0.1599	0.0772	2.1
Adenosine	C00212	components of purine metabolism	0.20605	0.25795	0.8
Arginine	C00062	amino acids and amino acid precursors	0.00075	0.00105	0.7

Aspartic Acid	C00049	amino acids and amino acid precursors	0.0917	0.08535	1.1
Biotin	C00120	vitamins	0.017	0.0189	0.9
CMP	C00055	components of pyrimidine metabolism	0.0008	0.00115	0.7
D-Ribose 5-Phosphate	C00117	components of carbon metabolism	0.0175	0.0172	1
Folate	C00504	vitamins	0.0095	0.00405	2.3
Glucose-6-Phosphate	C00092	components of carbon metabolism	0.0645	0.05115	1.3
Glutamine	C00064	amino acids and amino acid precursors	0.0026	0.0032	0.8
Glutammic Acid	C00025	amino acids and amino acid precursors	0.017	0.0166	1
GMP	C00144	components of purine metabolism	0.00015	0.00025	0.6
Histidine	C00135	amino acids and amino acid precursors	0.0035	0.0046	0.8

Isoleucine	C00407	amino acids and amino acid precursors	0.0245	0.016	1.5
Leucine	C00123	amino acids and amino acid precursors	0.0183	0.01025	1.8
Lysine	C00047	amino acids and amino acid precursors	0.00375	0.0038	1
Malonyl Coa	C00083	components of carbon metabolism	0.00635	0.0081	0.8
Methionine	C00073	amino acids and amino acid precursors	<0.0005	<0.0005	NA
Ornithine	C00077	amino acids and amino acid precursors	0.00415	0.0035	1.2
Oxalacetate	C00036	components of carbon metabolism	0.00058	0.00089	0.7
Riboflavin	C00255	vitamins	0.01125	0.01515	0.7
S-Adenosyl-Methionine	C00019	amino acids and amino acid precursors	<0.0005	<0.0005	NA

Serine	C00065	amino acids and amino acid precursors	0.0044	0.00285	1.5
Thiamine	C00378	vitamins	0.54975	0.59235	0.9
Thryptophan	C00078	amino acids and amino acid precursors	0.01525	0.01085	1.4
Tyrosine	C00082	amino acids and amino acid precursors	<0.0005	<0.0005	NA
UDP-N-Acetylmuramyl-Pentapeptide	C04702	components of peptidoglycan biosynthesis	0.0009	0.0009	1
UMP	C00105	components of pyrimidine metabolism	0.00095	0.0011	0.9
Undecyl Prodigiosin	C12023	antibiotics	0.00705	0.00515	1.4
Valine	C00183	amino acids and amino acid precursors	0.00445	0.00425	1
Vitamin B12	C05776	vitamins	0.00085	0.00045	1.9
1,3-Bisphospho-D-Glycerate	C00236	components of carbon metabolism	<0.00001	<0.00001	NA

2-Oxoglutarate	C00026	components of carbon metabolism	<0.00001	<0.00001	NA
3-Phosphoglycerate	C00197	components of carbon metabolism	<0.00001	<0.00001	NA
5-Phospho-Alpha-D-Ribose 1-Diphosphate	C00119	components of carbon metabolism	<0.00001	<0.00001	NA
Acetyl Phosphate	C00227	components of carbon metabolism	<0.00001	<0.00001	NA
Adenine	C00147	components of purine metabolism	<0.00001	<0.00001	NA
ADP	C00008	components of purine metabolism	<0.00001	<0.00001	NA
Alanine	C00041	amino acids and amino acid precursors	<0.00001	<0.00001	NA
AMP	C00020	components of purine metabolism	<0.00001	<0.00001	NA
Anthranilate	C00108	amino acids and amino acid precursors	<0.00001	<0.00001	NA

Calcium-Dependent Antibiotic	C12024	antibiotics	<0.00001	<0.00001	NA
Asparagine	C00152	amino acids and amino acid precursors	<0.00001	<0.00001	NA
ATP	C00002	components of purine metabolism	<0.00001	<0.00001	NA
Camp	C00575	components of purine metabolism	<0.00001	<0.00001	NA
Cgmp	C00942	components of purine metabolism	<0.00001	<0.00001	NA
Chorismate	C00251	amino acids and amino acid precursors	<0.00001	<0.00001	NA
Cis-Aconitate	C00417	components of carbon metabolism	<0.00001	<0.00001	NA
Citrate	C00158	components of carbon metabolism	<0.00001	<0.00001	NA
Citrulline	C00327	amino acids and amino acid precursors	<0.00001	<0.00001	NA

Coenzyme A	C00010	components of carbon metabolism	<0.00001	<0.00001	NA
CTP	C00063	components of pyrimidine metabolism	<0.00001	<0.00001	NA
FAD	C00016	coenzyme	<0.00001	<0.00001	NA
D-Fructose 6-Phosphate	C00085	components of carbon metabolism	<0.00001	<0.00001	NA
D-Fructose 1,6-Bisphosphate	C00354	components of carbon metabolism	<0.00001	<0.00001	NA
Fumarate	C00122	components of carbon metabolism	<0.00001	<0.00001	NA
Gamma-Butyrolactone	C01770	other	<0.00001	<0.00001	NA
GDP	C00035	components of purine metabolism	<0.00001	<0.00001	NA
D-Glucosamine 6-Phosphate	C00352	amino acids and amino acid precursors	<0.00001	<0.00001	NA
Glutaconate	C02214	other	<0.00001	<0.00001	NA

D-Glyceraldehyde 3-Phosphate	C00118	components of carbon metabolism	<0.00001	<0.00001	NA
Glycine	C00037	amino acids and amino acid precursors	<0.00001	<0.00001	NA
GTP	C00044	components of purine metabolism	<0.00001	<0.00001	NA
Guanine	C00242	components of purine metabolism	<0.00001	<0.00001	NA
Homocysteine	C00155	amino acids and amino acid precursors	<0.00001	<0.00001	NA
IMP	C00130	components of purine metabolism	<0.00001	<0.00001	NA
Inosine	C00294	components of purine metabolism	<0.00001	<0.00001	NA
ITP	C00081	components of purine metabolism	<0.00001	<0.00001	NA
Malate	C00149	components of carbon metabolism	<0.00001	<0.00001	NA

Methylmalonyl Coa	C00083	components of carbon metabolism	<0.00001	<0.00001	NA
Methyl Malonate	C02170	components of pyrimidine metabolism	<0.00001	<0.00001	NA
N-Acetyl-D-Glucosamine	C00140	components of peptidoglycan biosynthesis	<0.00001	<0.00001	NA
N-Acetyl-Muramic	C02713	components of peptidoglycan biosynthesis	<0.00001	<0.00001	NA
NAD	C00003	coenzyme	<0.00001	<0.00001	NA
NADH	C00004	coenzyme	<0.00001	<0.00001	NA
NADPH	C00005	coenzyme	<0.00001	<0.00001	NA
Oxalate	C00209	components of purine metabolism	<0.00001	<0.00001	NA
Oxidized Glutathione	C00127	other	<0.00001	<0.00001	NA
Phenylpyruvate	C00166	amino acids and amino acid precursors	<0.00001	<0.00001	NA
Phosphoenolpyruvate	C00074	components of carbon metabolism	<0.00001	<0.00001	NA

Polyphosphate	C00404	other	<0.00001	<0.00001	NA
Proline	C00148	amino acids and amino acid precursors	<0.00001	<0.00001	NA
Propionyl_Coa	C00100	other	<0.00001	<0.00001	NA
Pyruvate	C00022	components of carbon metabolism	<0.00001	<0.00001	NA
Reduced Glutathione	C00051	other	<0.00001	<0.00001	NA
S-Adenosyl-Homocysteine	C00021	amino acids and amino acid precursors	<0.00001	<0.00001	NA
Succinate	C00042	components of carbon metabolism	<0.00001	<0.00001	NA
Succinyl_Coa	C00091	components of carbon metabolism	<0.00001	<0.00001	NA
UDP	C00015	components of pyrimidine metabolism	<0.00001	<0.00001	NA
UDP-Glucuronate	C00167	other	<0.00001	<0.00001	NA
UDP-N-Acetylglucosamine	C00043	components of peptidoglycan biosynthesis	<0.00001	<0.00001	NA

Uracil	C00106	components of pyrimidine metabolism	<0.00001	<0.00001	NA
Uridine	C00299	components of pyrimidine metabolism	<0.00001	<0.00001	NA
UTP	C00075	components of pyrimidine metabolism	<0.00001	<0.00001	NA
Thymine	C00178	components of pyrimidine metabolism	<0.00001	<0.00001	NA

[a] Fold change for each metabolite was calculated by normalizing the amount of the F3 fraction to that of the F4 counterpart. NA: not applicable.

Table S4. Results of untargeted metabolomics from LC-MS/MS analysis of *S. coelicolor* MVs. Reported are the compound name (whenever available), and the corresponding retention time, experimental and theorical mass value and calculated formula of identified/non-identified metabolites. ND, not determined.

	Compound	t_R (min)	Theor. Mass	Exp. Mass (m/z)	Formula
1	Unknown 1	4.67	ND	775.8650 $[M+2H]^{2+}$<br>773.8514 $[M-2H]^{2-}$	ND
2	Unknown 2	4.89	ND	1422.8126 $[M+H]^{+}$<br>1420.8195 $[M-H]^{-}$	ND
3	Unknown 3	5.31	ND	1177.2973 $[M+2H]^{2+}$	ND
4	Unknown 4	9.43	228.1958 $[M+H]^{+}$	228.1966 $[M+H]^{+}$	$C_{13}H_{25}NO_2$
5	Unknown 5	9.54	ND	795.2450 $[M+Na]^{+}$	ND
6	Furaquinocin D	10.08	431.1711 $[M+FA]^{-}$	431.1700 $[M+FA]^{-}$	$C_{22}H_{26}O_6$
7	Medelamine C	10.32	272.2584 $[M+H]^{+}$	272.2583 $[M+H]^{+}$	$C_{16}H_{33}NO_2$
8	C16 Sphingosine	10.83	272.2584 $[M+H]^{+}$	272.2578 $[M+H]^{+}$	$C_{16}H_{33}NO_2$
9	Unknown 6	11.09	ND	616.1776 $[M+H]^{+}$	ND
10	Streptorubin B	11.86	392.2696 $[M+H]^{+}$	392.2724 $[M+H]^{+}$	$C_{25}H_{33}N_3O$
11	Unknown 7	12.05	ND	847.4727 $[M+H]^{+}$	ND
12	Undecylprodigiosin	12.49	394.2853 $[M+H]^{+}$	394.2856 $[M+H]^{+}$	$C_{25}H_{35}N_3O$

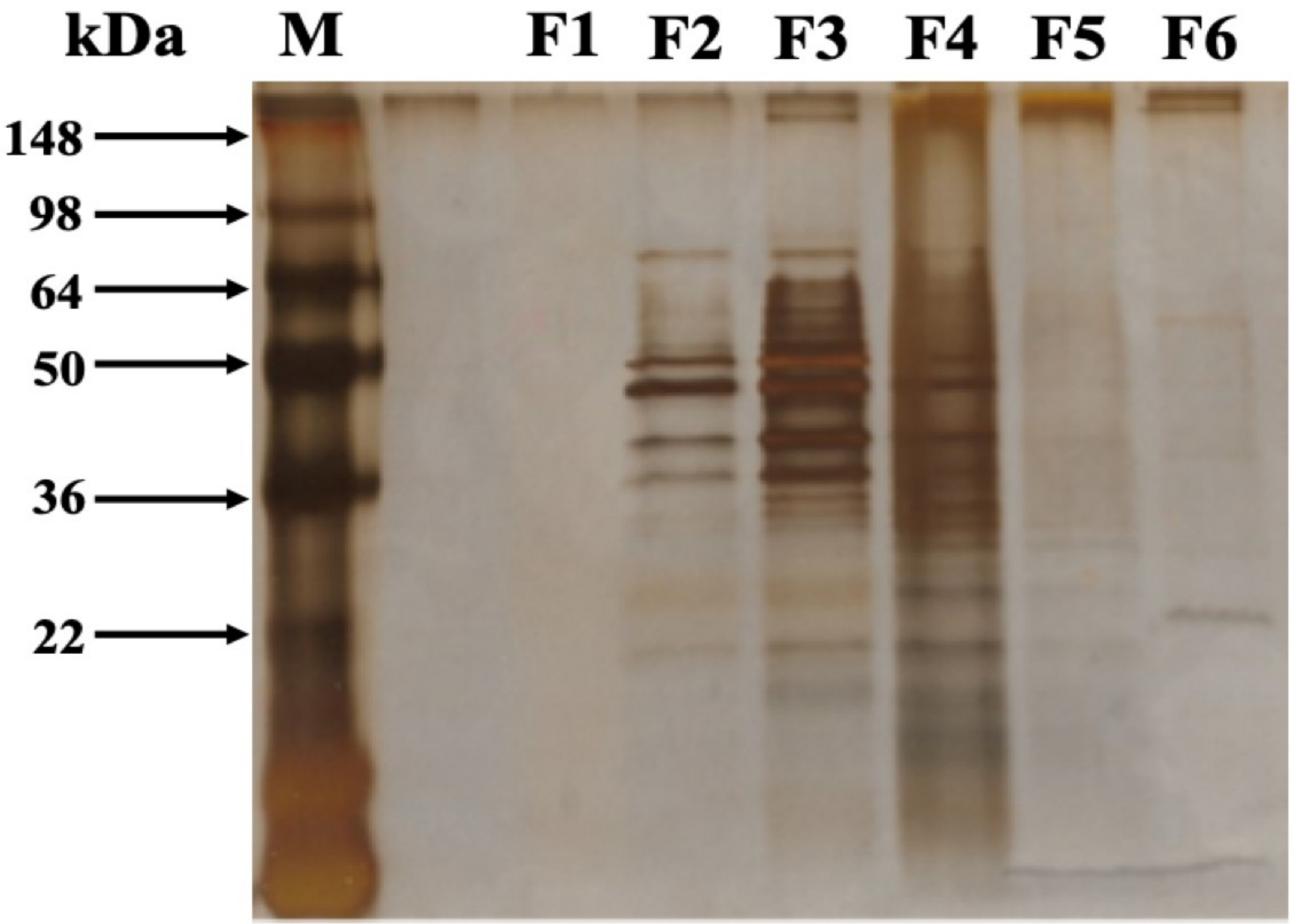

Figure S1: SDS-PAGE profile of Optiprep gradient fractions (F1-F6) of *S. coelicolor* supernatant. This figure shows a portion of the MV samples independently subjected to SDS-PAGE combined with proteomic and bioinformatic analyses.

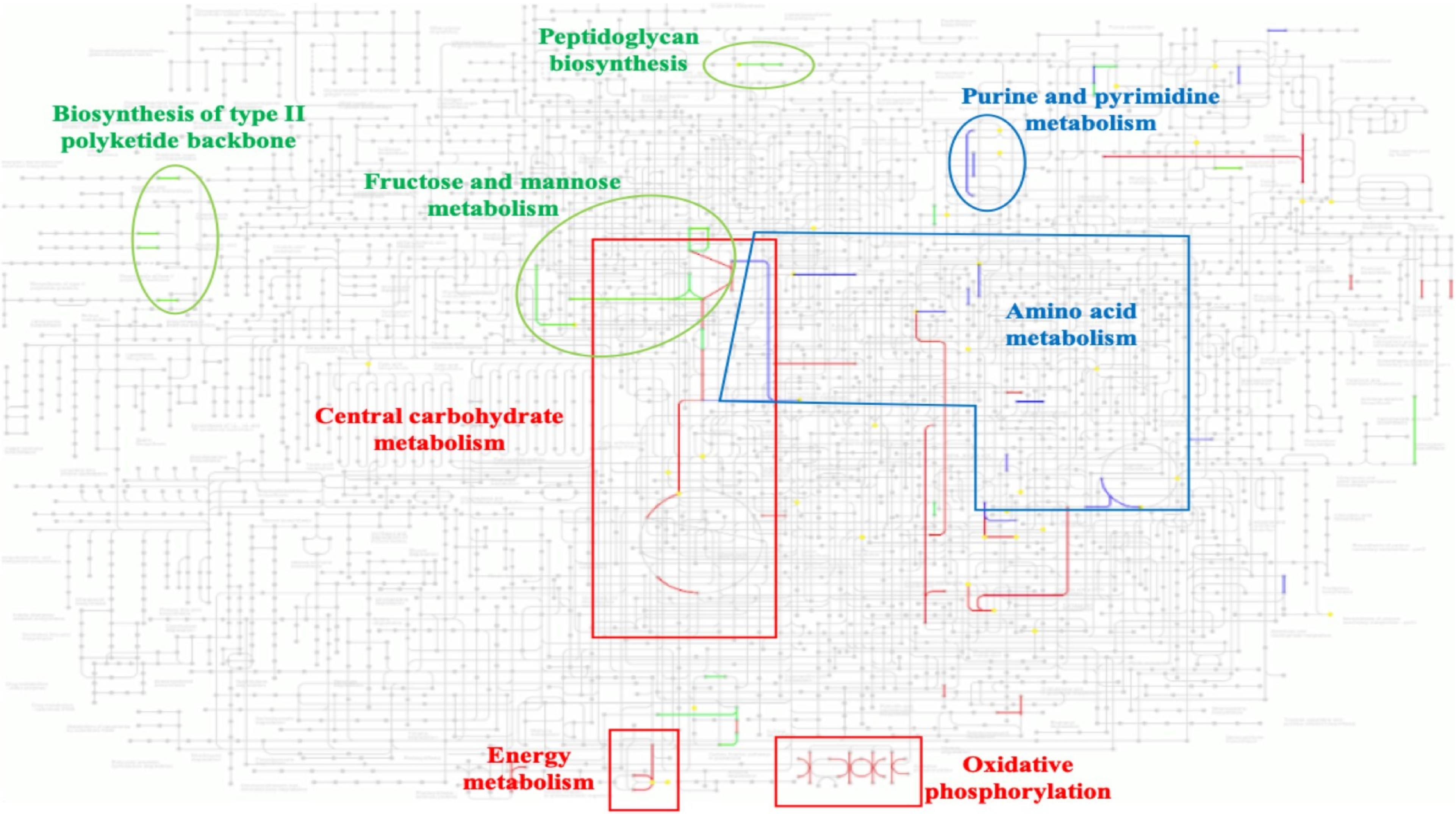

Figure S2: Combined KEGG proteomic and metabolic map of *S. coelicolor* MVs. F3 MVs-specific pathways are shown in green; F4 MVs-specific pathways are shown in blue; common pathways are shown in red, while metabolites are shown in yellow.

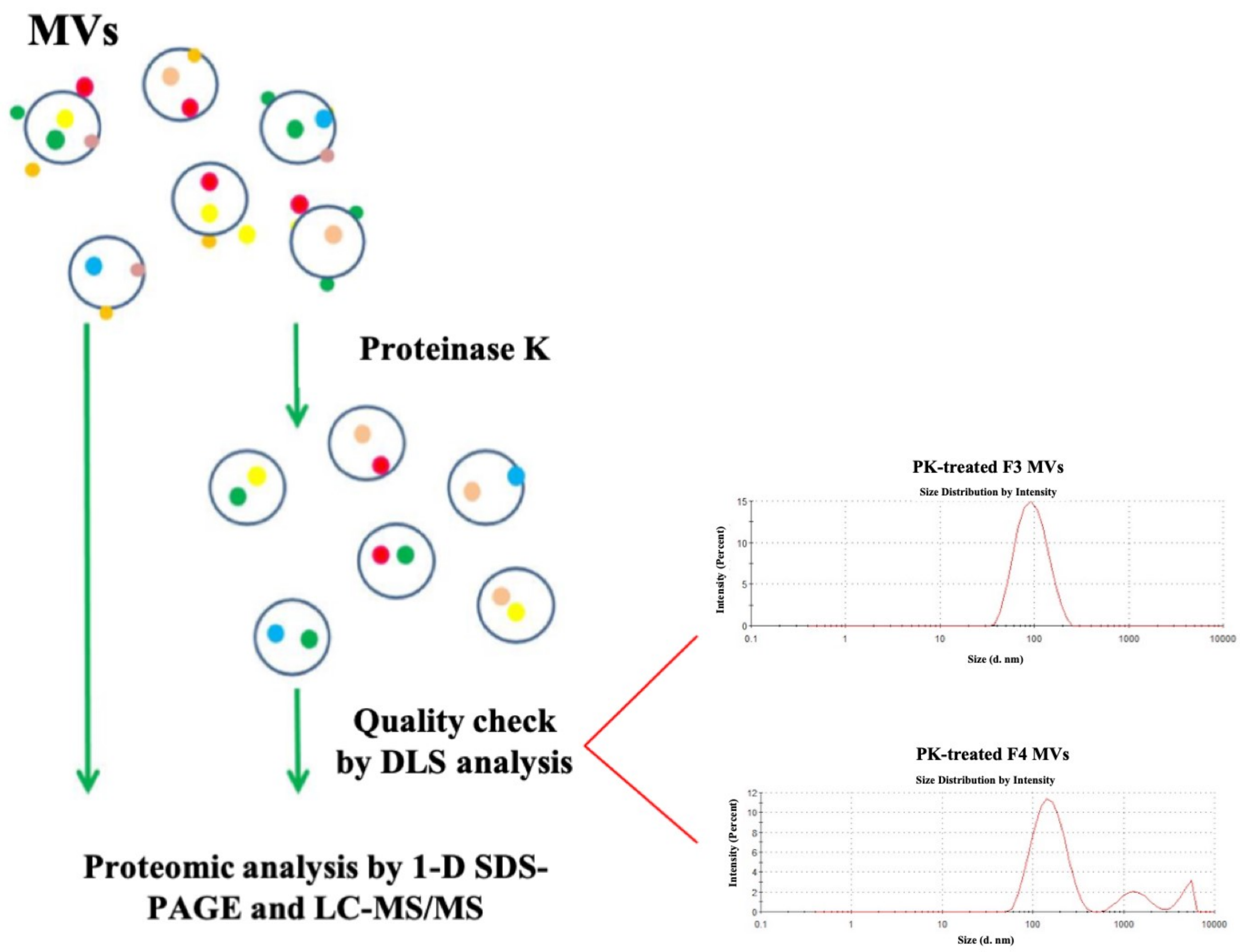

Figure S3: Diagram showing the experimental strategy to identify the luminal proteome of *S. coelicolor* MVs. MVs from fraction F3 and F4 were treated with proteinase K from *Tritirachium album* (PK). PK-treated and untreated MVs were analyzed in parallel by combined SDS-PAGE, proteomics and bioinformatics. DLS analysis revealed that PK treatment did not alter the vesicles.
